# Supplementary material for: The Effect of a Dominant Inhibitory p53 Protein on Stress Responses Induced by Toxic and Non-Toxic Concentrations of Anisomycin in PC12 Cells
Source: Biology (Basel). 2025 Nov 21;14(12):1634. doi: 10.3390/biology14121634 (PMC12729296; doi:10.3390/biology14121634)

Supplementary Figure 1. Original blots of Figure 1A.

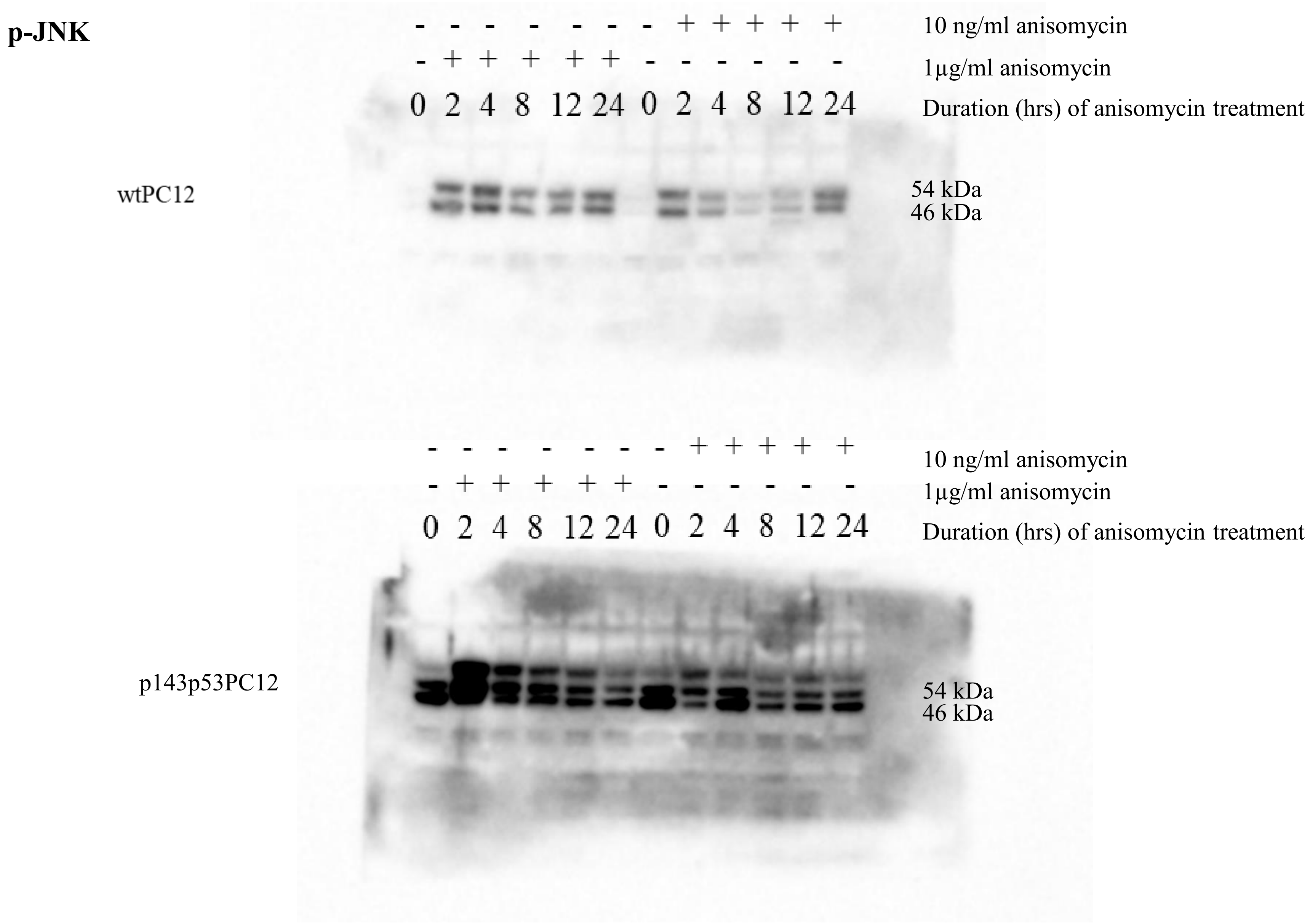

Supplementary Figure 1. Original blots of Figure 1A.

**JNK**

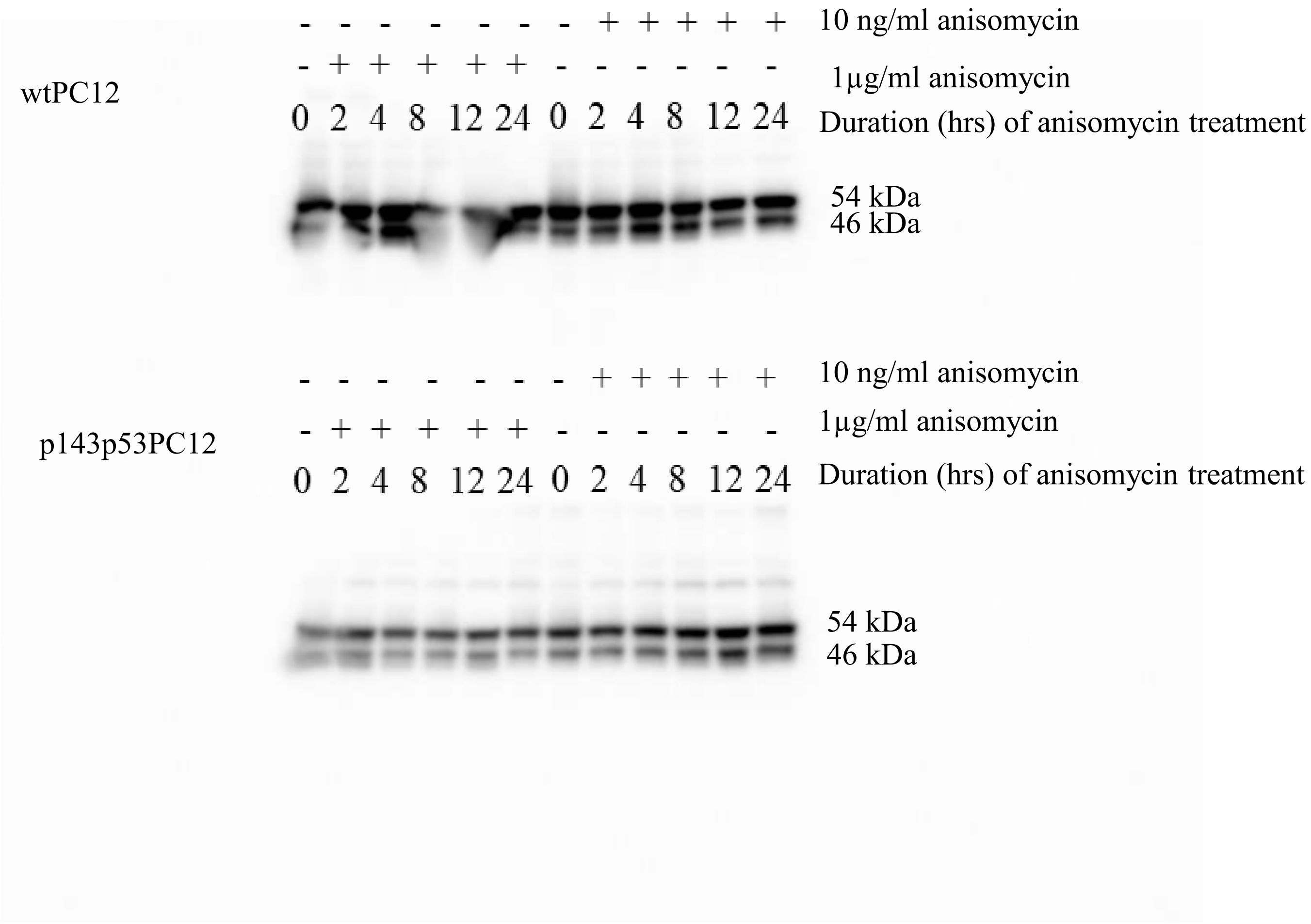

Supplementary Figure 1. Original blots of Figure 1A.

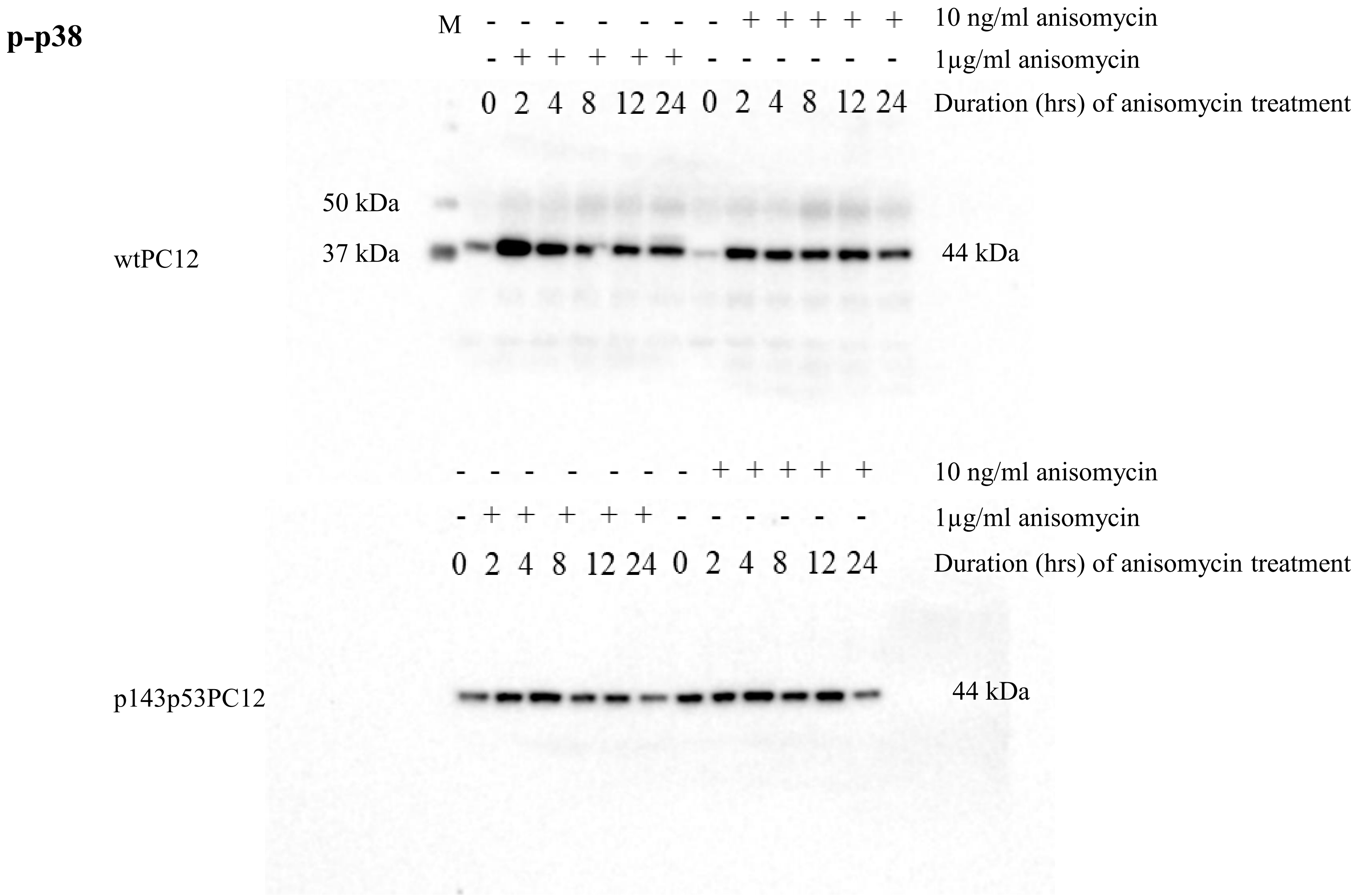

Supplementary Figure 1. Original blots of Figure 1A.

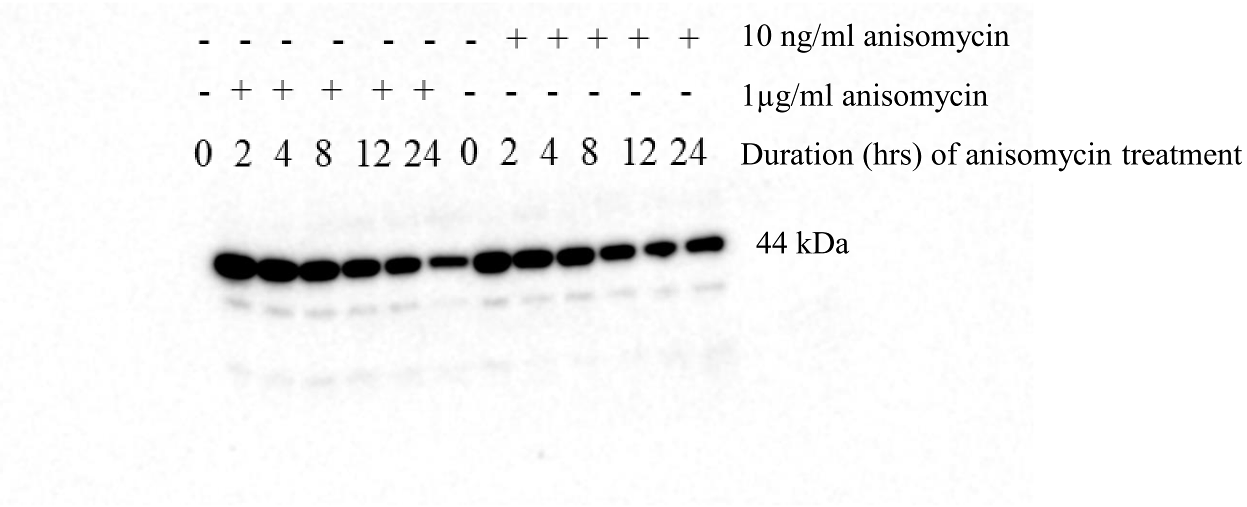


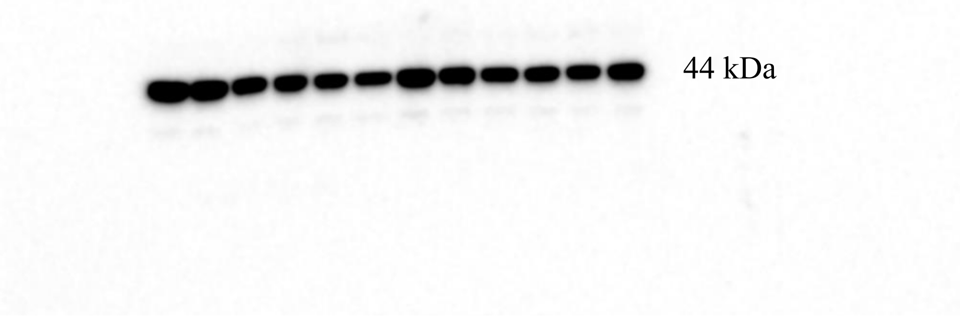

Supplementary Figure 1. Original blots of Figure 1A.

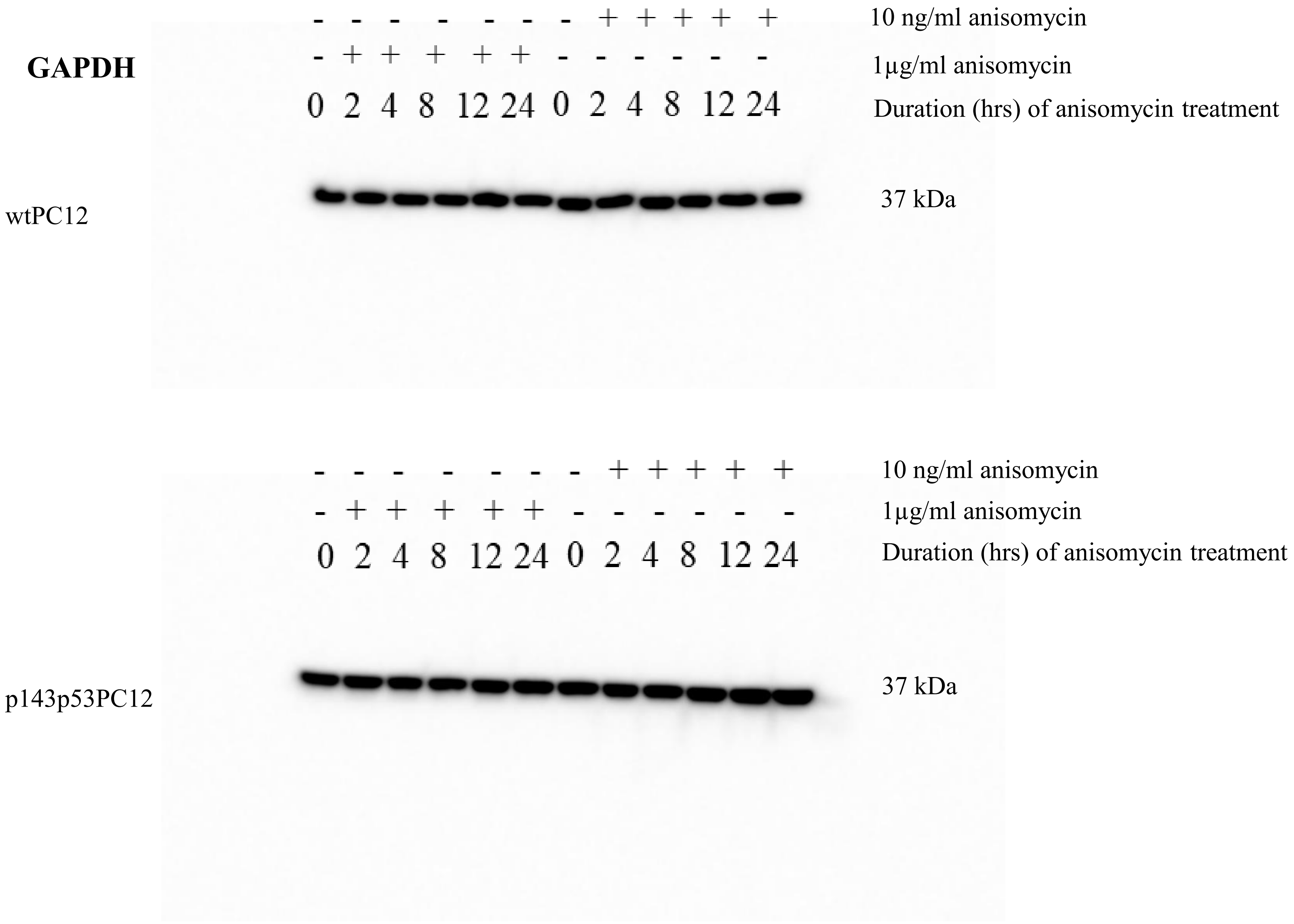

Supplementary Figure 1. Original blots of Figure 1A.

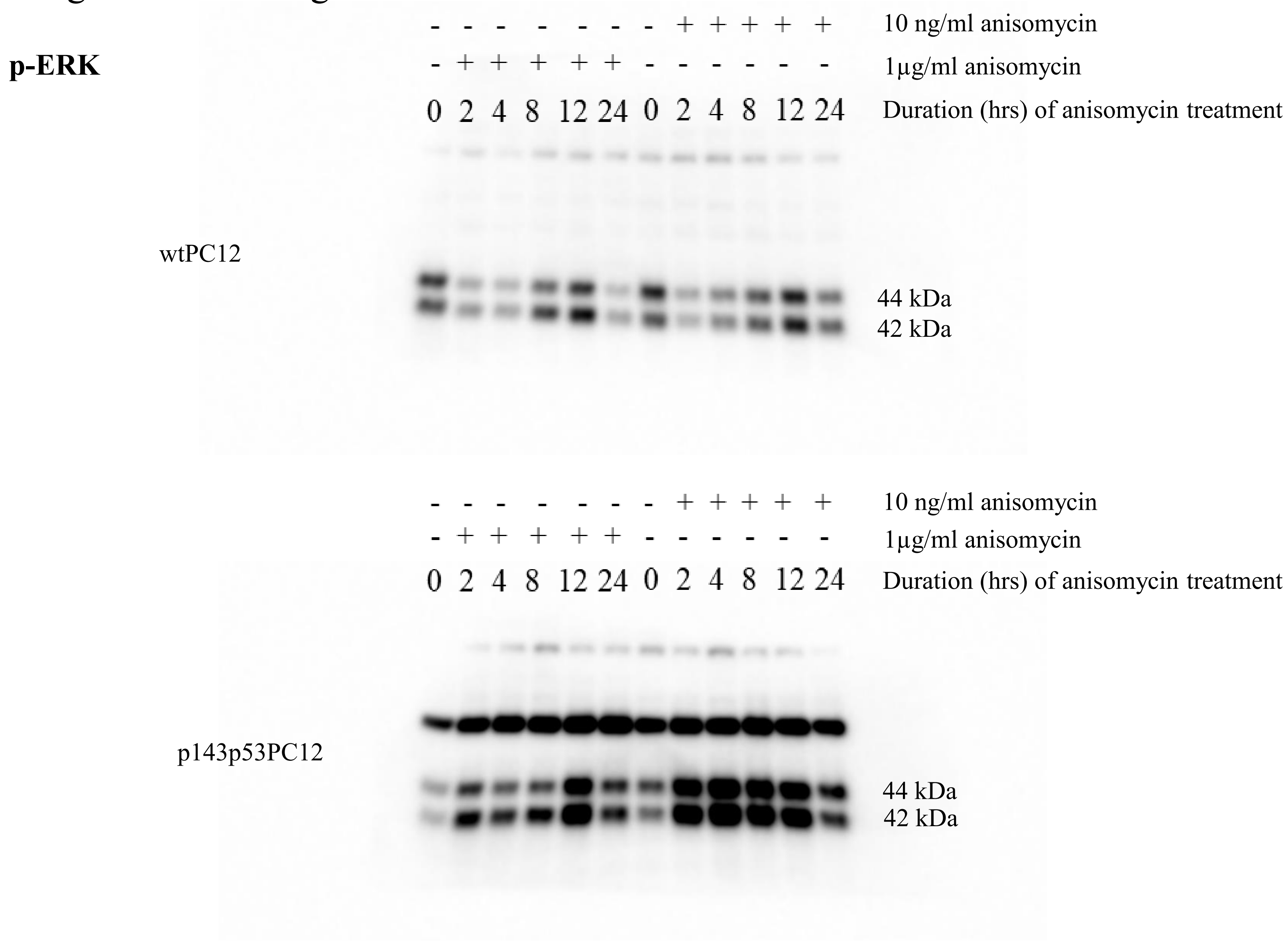

Supplementary Figure 1. Original blots of Figure 1A.

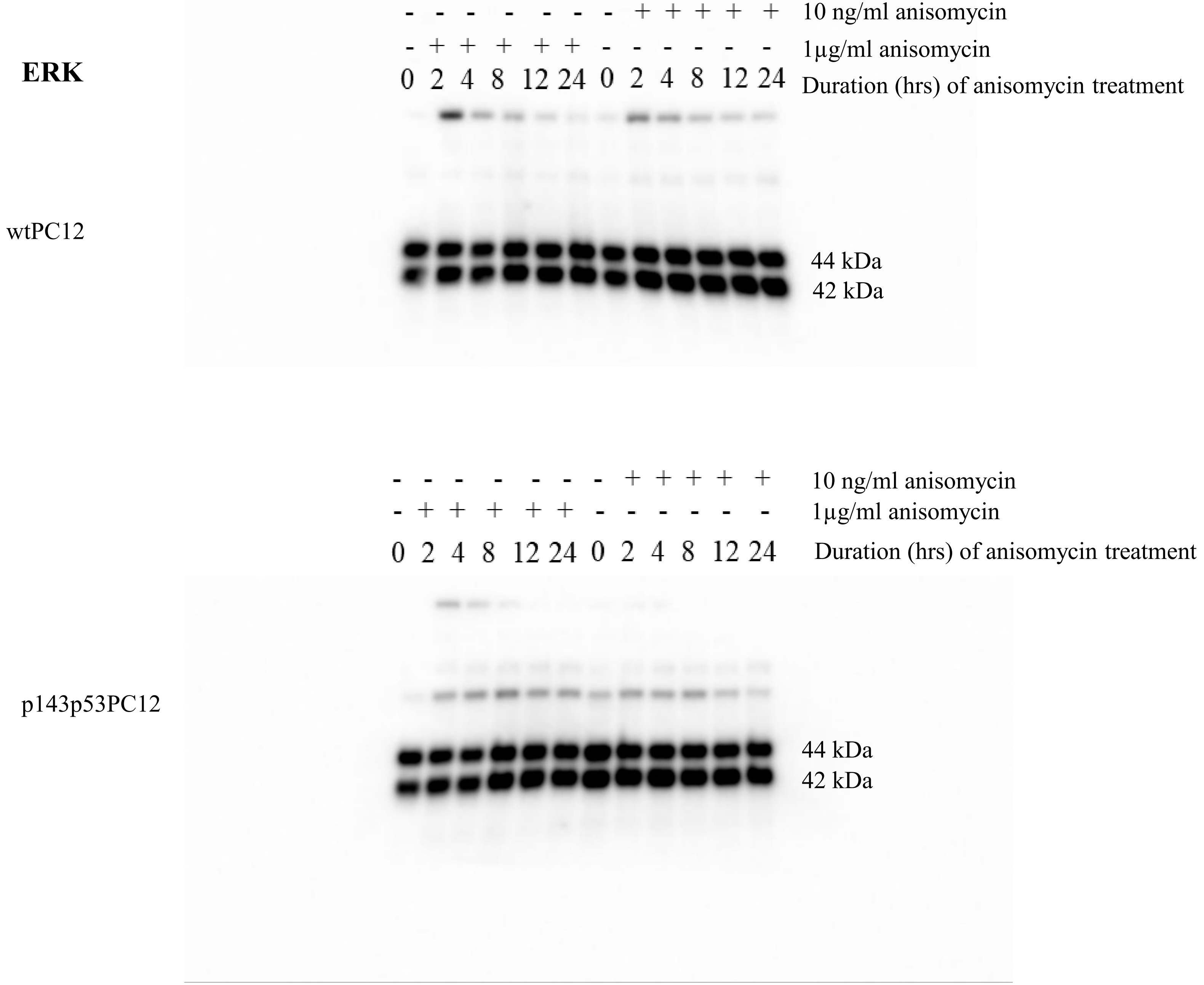

Supplementary Figure 1. Original blots of Figure 1B.

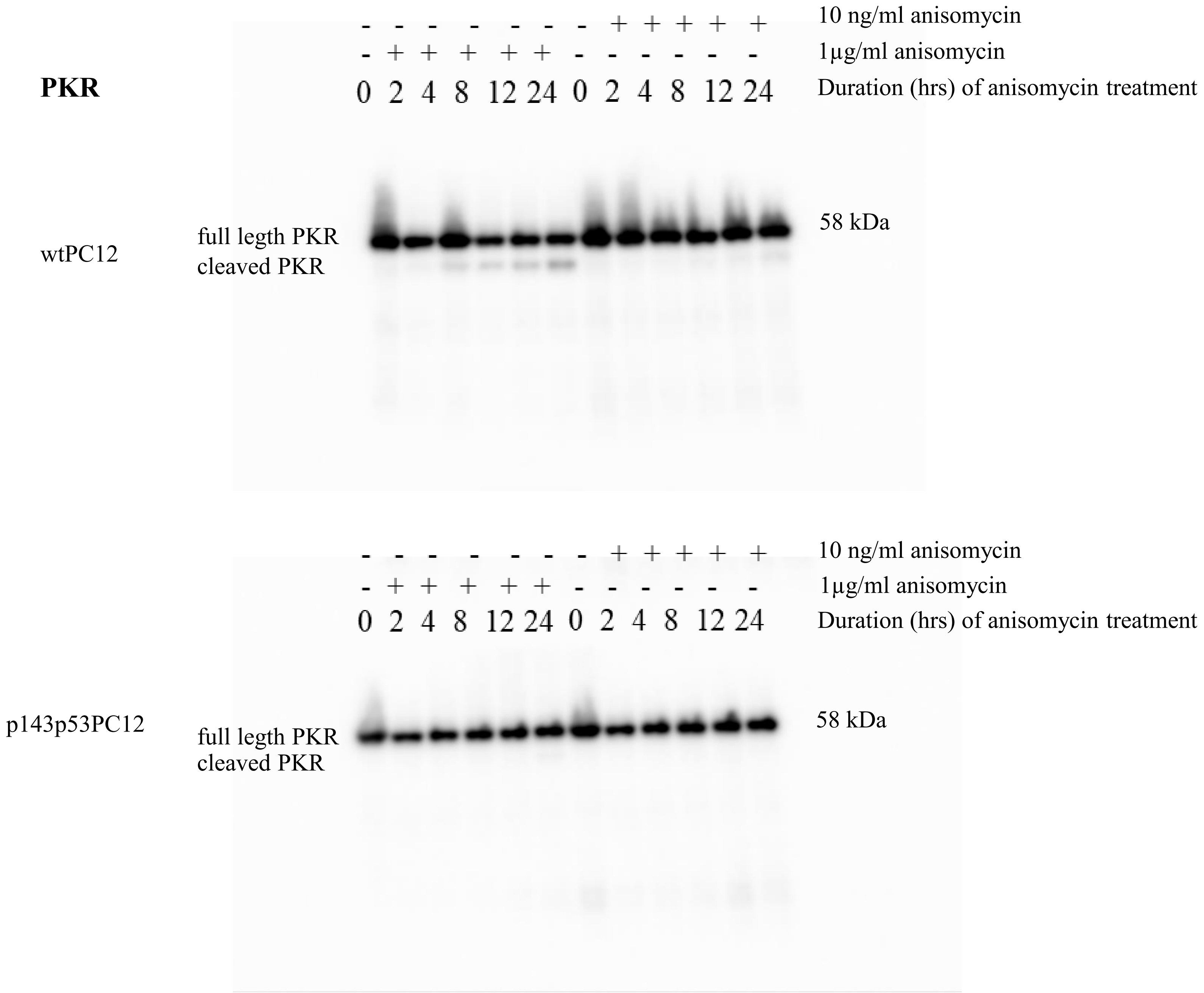

Supplementary Figure 1. Original blots of Figure 1B. (Kodak)

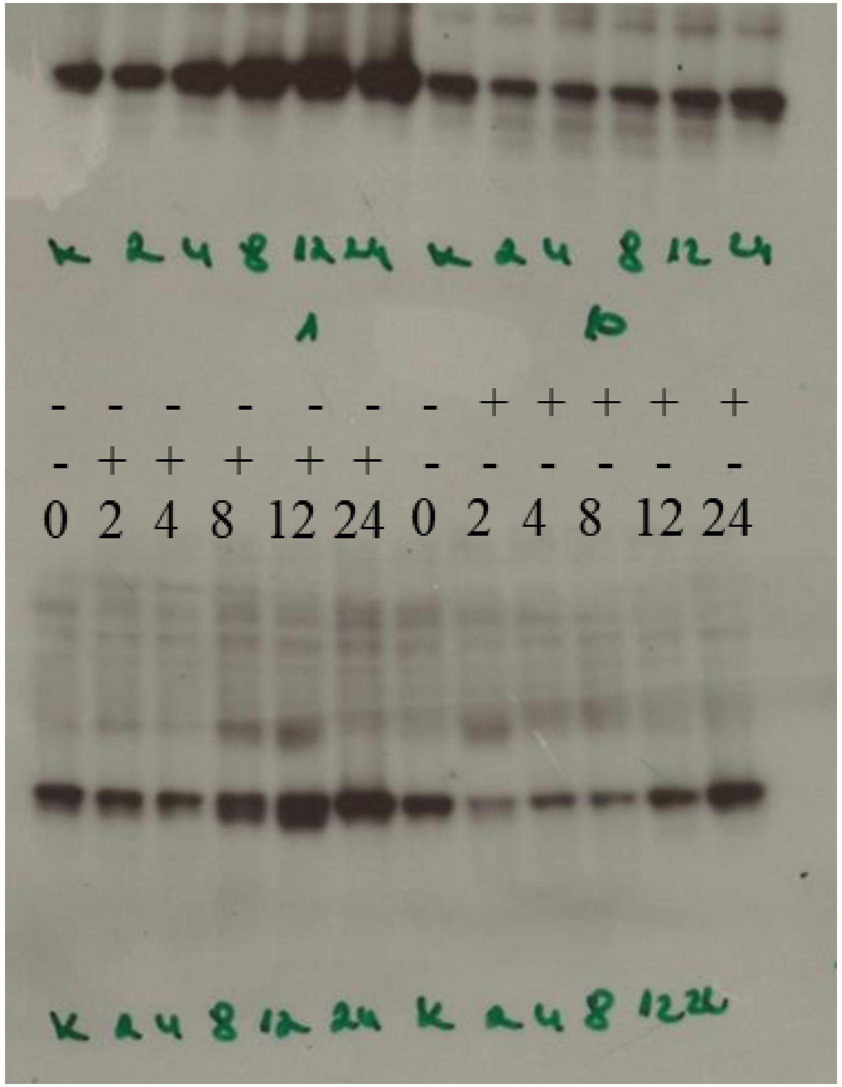

Supplementary Figure 1. Original blots of Figure 1B.

**eIF2α**

- - - - - - - + + + + + 10 ng/ml anisomycin

- + + + + + - - - - - - 1µg/ml anisomycin

0 2 4 8 12 24 0 2 4 8 12 24 Duration (hrs) of anisomycin treatment

wtPC12

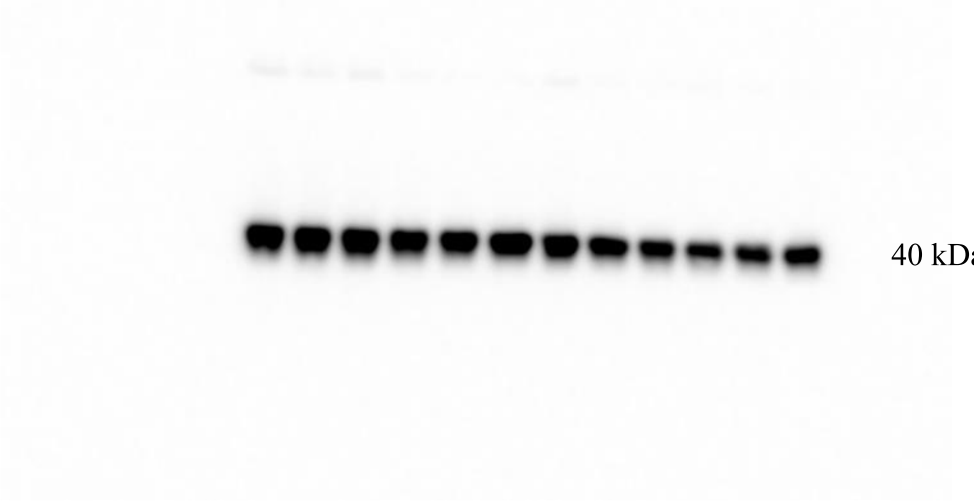

40 kDa

- - - - - - - + + + + + 10 ng/ml anisomycin

- + + + + + - - - - - - 1µg/ml anisomycin

0 2 4 8 12 24 0 2 4 8 12 24 Duration (hrs) of anisomycin treatment

p143p53PC12

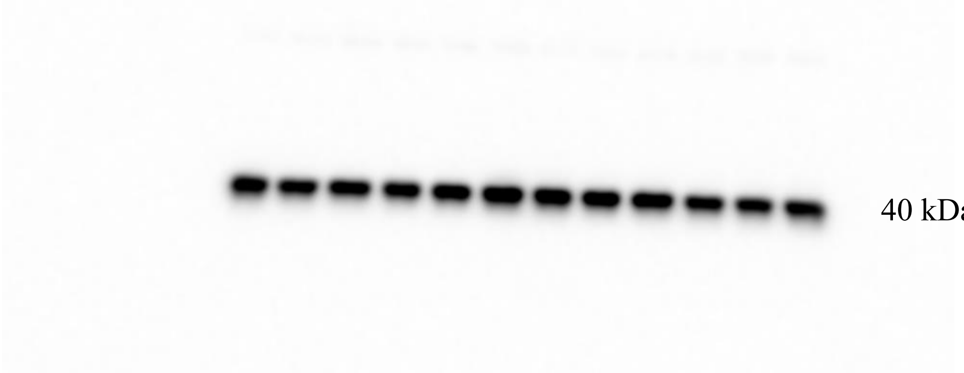

40 kDa

Supplementary Figure 2. Original blots of Figure 2. (Kodak)

**Cleaved caspase-9**

| | M | 0 | 2 | 4 | 8 | 12 | 24 | 0 | 2 | 4 | 8 | 12 | 24 |
|---|---|---|---|---|---|---|---|---|---|---|---|---|---|
| 10 ng/ml anisomycin | | - | - | - | - | - | - | - | + | + | + | + | + |
| 1μg/ml anisomycin | | - | + | + | + | + | + | - | - | - | - | - | - |

M 0 2 4 8 12 24 0 2 4 8 12 24 Duration (hrs) of anisomycin treatment

wtPC12

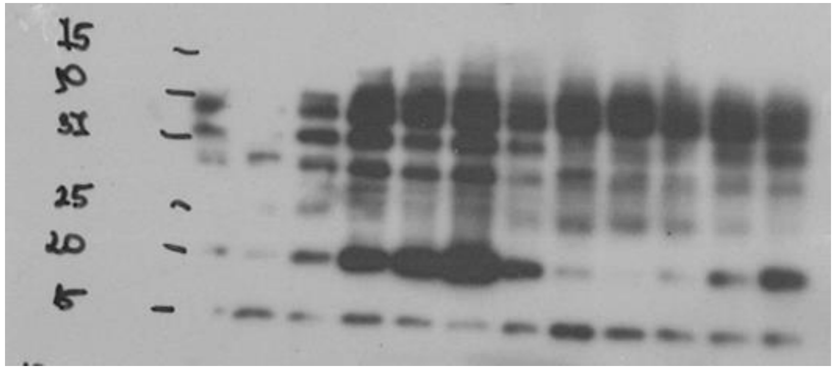

51 kDa (full)
40 kDa (full)
37,5 kDa (cleaved)
17 kDa (cleaved)

| | M | 0 | 2 | 4 | 8 | 12 | 24 | 0 | 2 | 4 | 8 | 12 | 24 |
|---|---|---|---|---|---|---|---|---|---|---|---|---|---|
| 10 ng/ml anisomycin | | - | - | - | - | - | - | - | + | + | + | + | + |
| 1μg/ml anisomycin | | - | + | + | + | + | + | - | - | - | - | - | - |

M 0 2 4 8 12 24 0 2 4 8 12 24 Duration (hrs) of anisomycin treatment

p143p53PC12

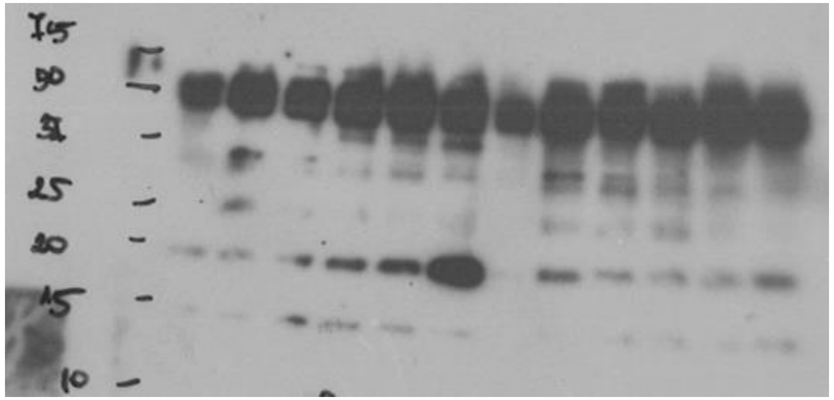

51 kDa (full)
40 kDa (full)
37,5 kDa (cleaved)
17 kDa (cleaved)

Supplementary Figure 2. Original blots of Figure 2.

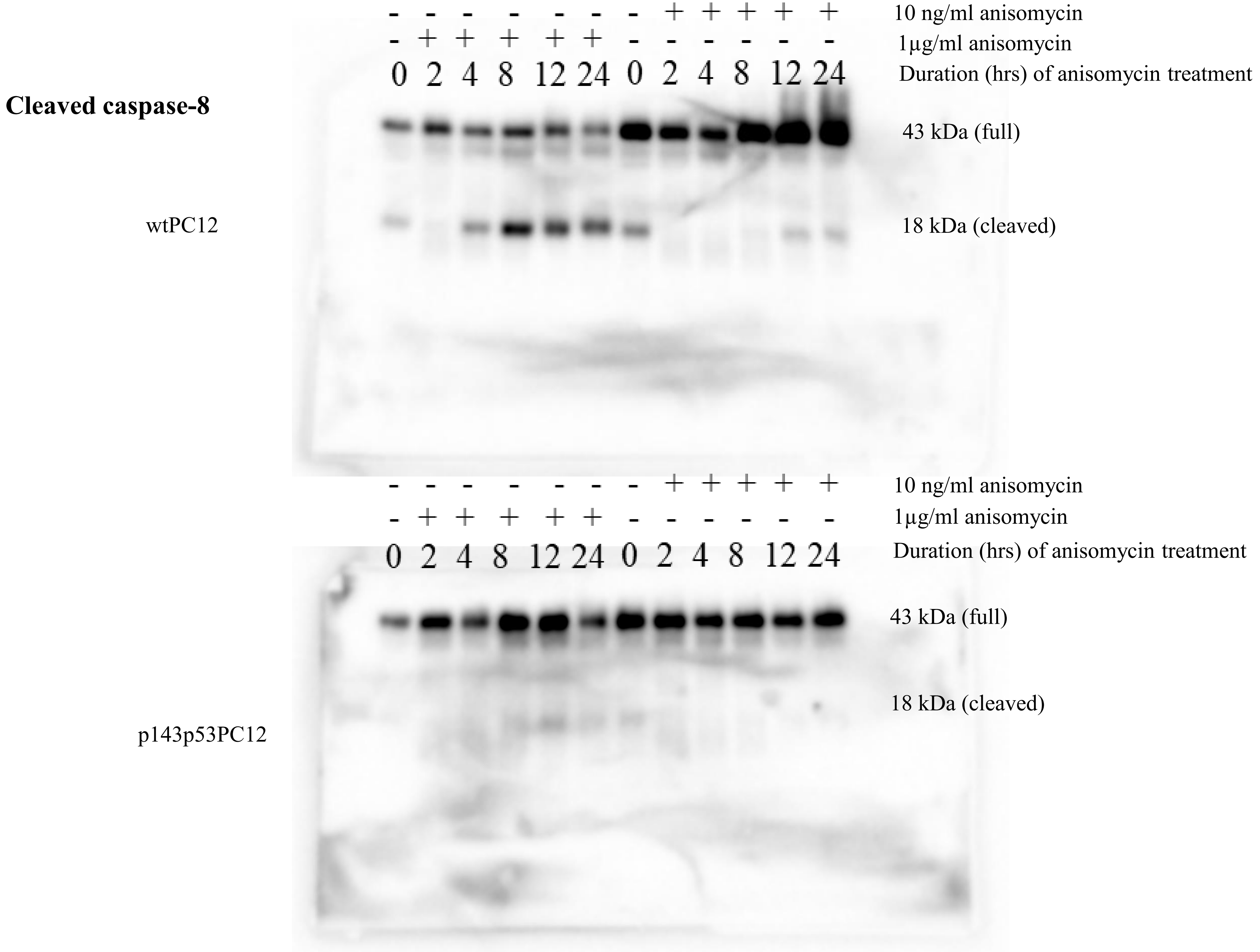

Supplementary Figure 2. Original blots of Figure 2. (Kodak)

**Cleaved caspase-3**

| | | | | | | | | | | | | |
|---|---|---|---|---|---|---|---|---|---|---|---|---|
| - | - | - | - | - | - | - | + | + | + | + | + | 10 ng/ml anisomycin |
| - | + | + | + | + | + | - | - | - | - | - | - | 1μg/ml anisomycin |
| 0 | 2 | 4 | 8 | 12 | 24 | 0 | 2 | 4 | 8 | 12 | 24 | Duration (hrs) of anisomycin treatment |

wtPC12

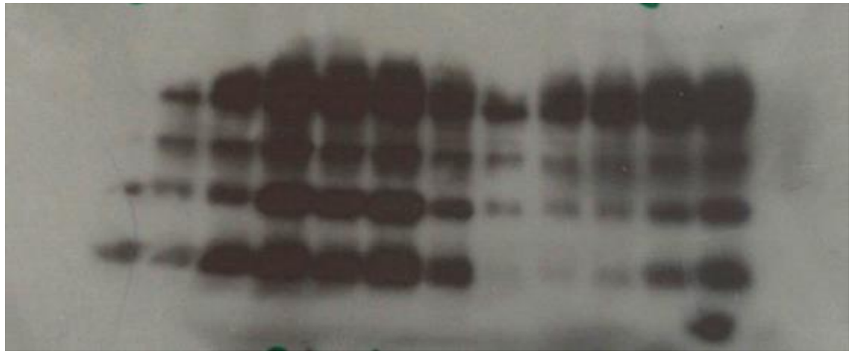

32 kDa (full)

19 kDa (cleaved)

| | | | | | | | | | | | | |
|---|---|---|---|---|---|---|---|---|---|---|---|---|
| - | - | - | - | - | - | - | + | + | + | + | + | 10 ng/ml anisomycin |
| - | + | + | + | + | + | - | - | - | - | - | - | 1μg/ml anisomycin |
| 0 | 2 | 4 | 8 | 12 | 24 | 0 | 2 | 4 | 8 | 12 | 24 | Duration (hrs) of anisomycin treatment |

p143p53PC12

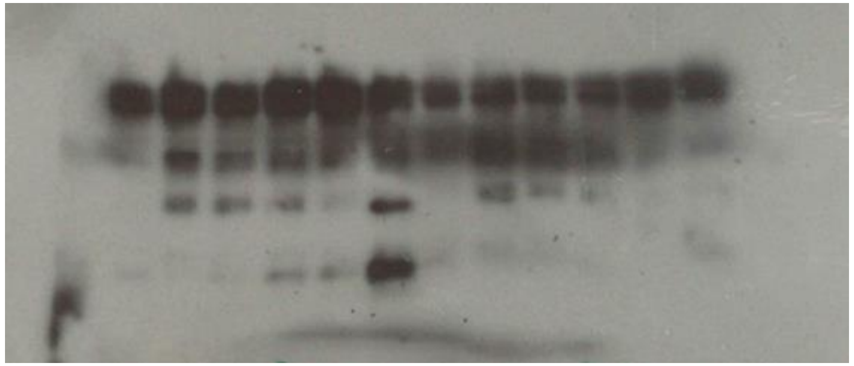

32 kDa (full)

19 kDa (cleaved)

Supplementary Figure 2. Original blots of Figure 2.

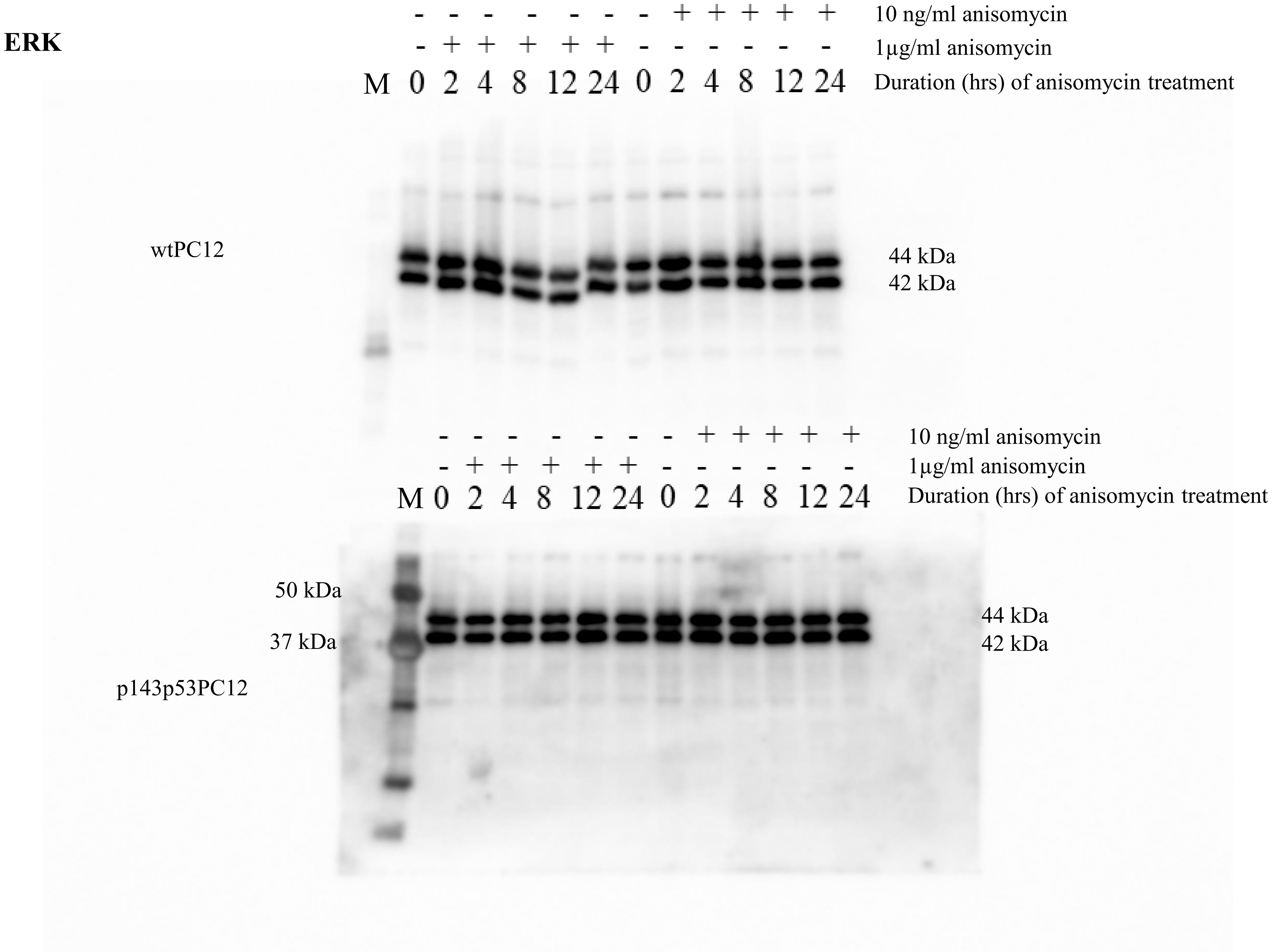

Supplementary Figure 3. Original blots of Figure 3.

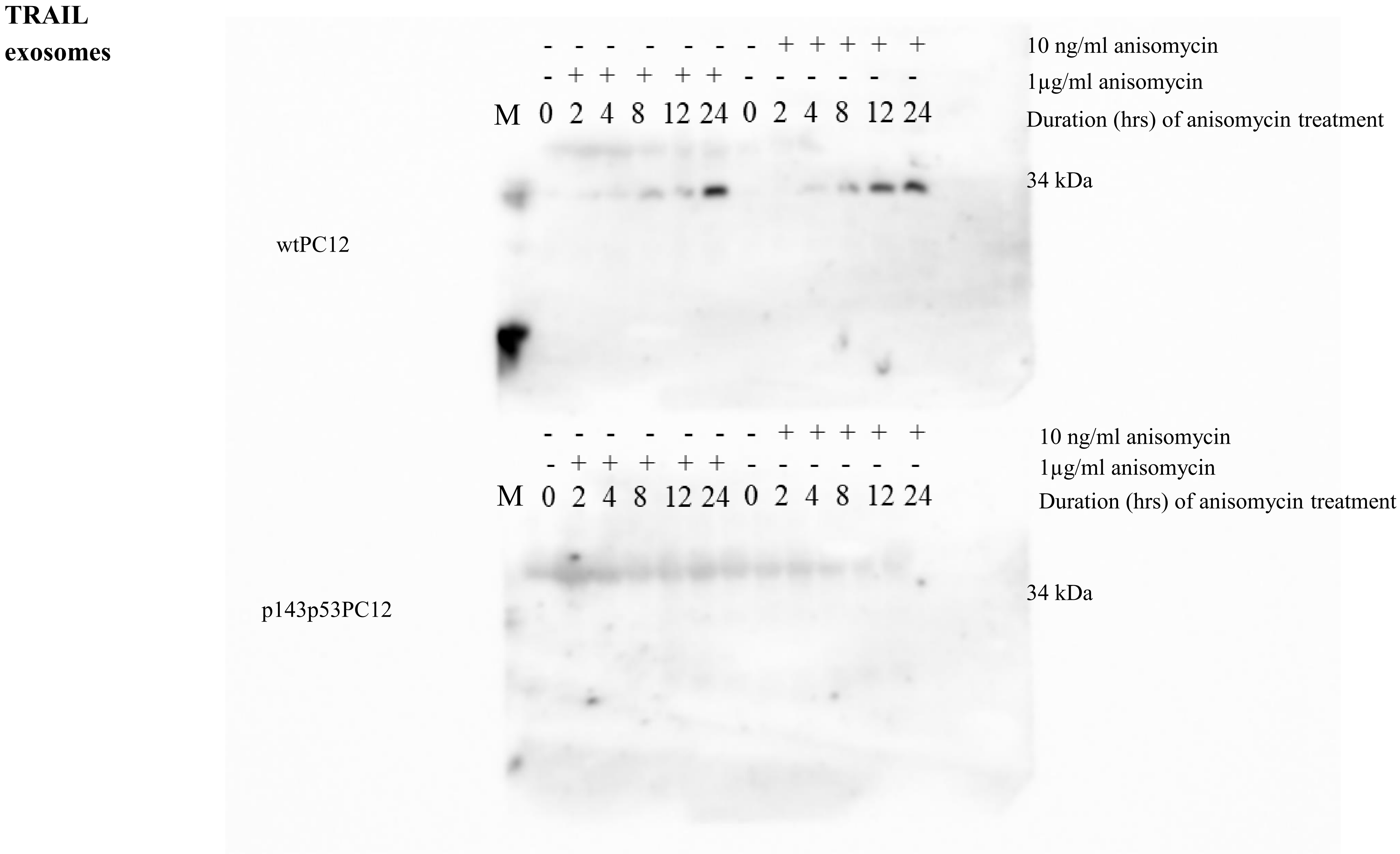

Supplementary Figure 3. Original blots of Figure 3.

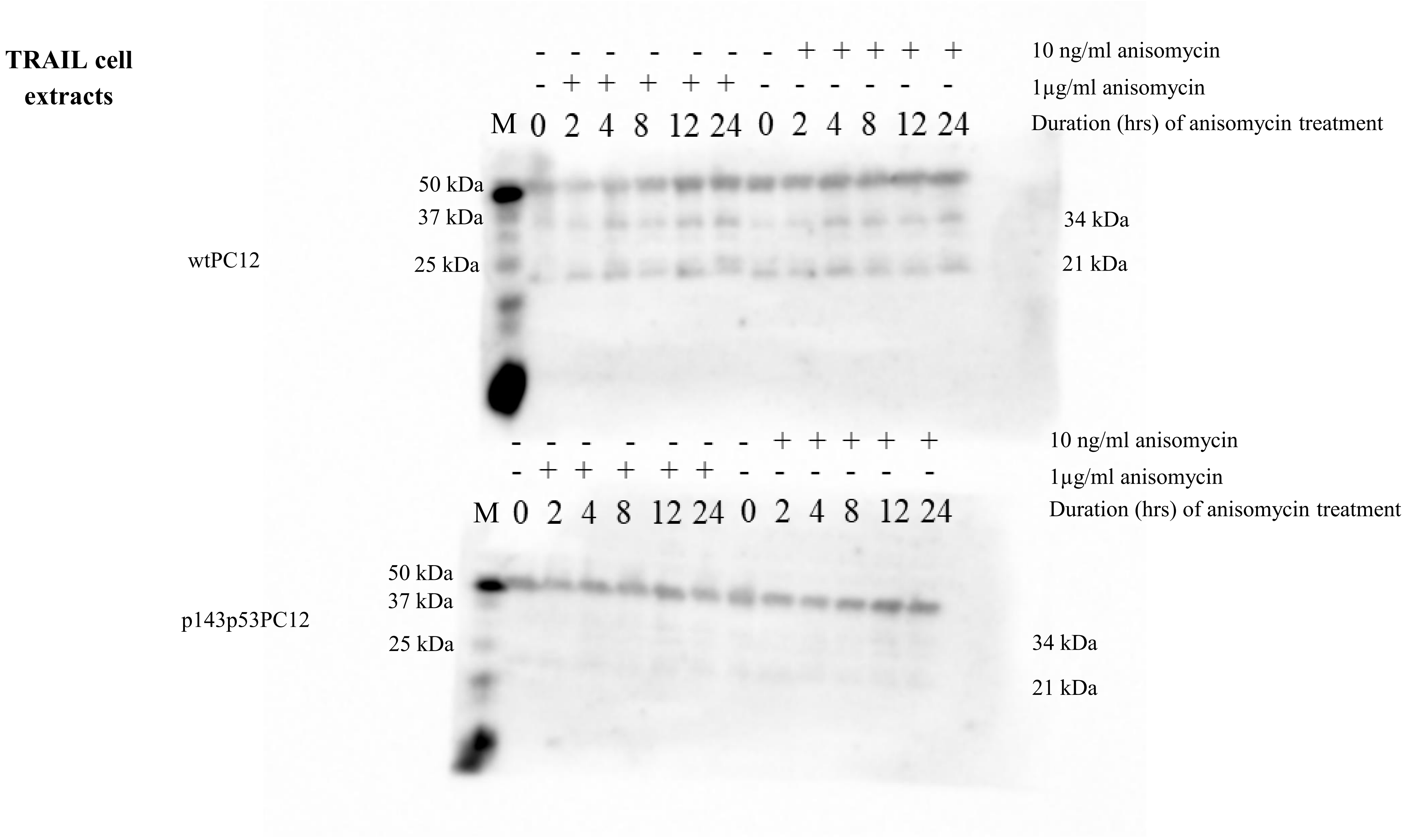

Supplementary Figure 3. Original blots of Figure 3.

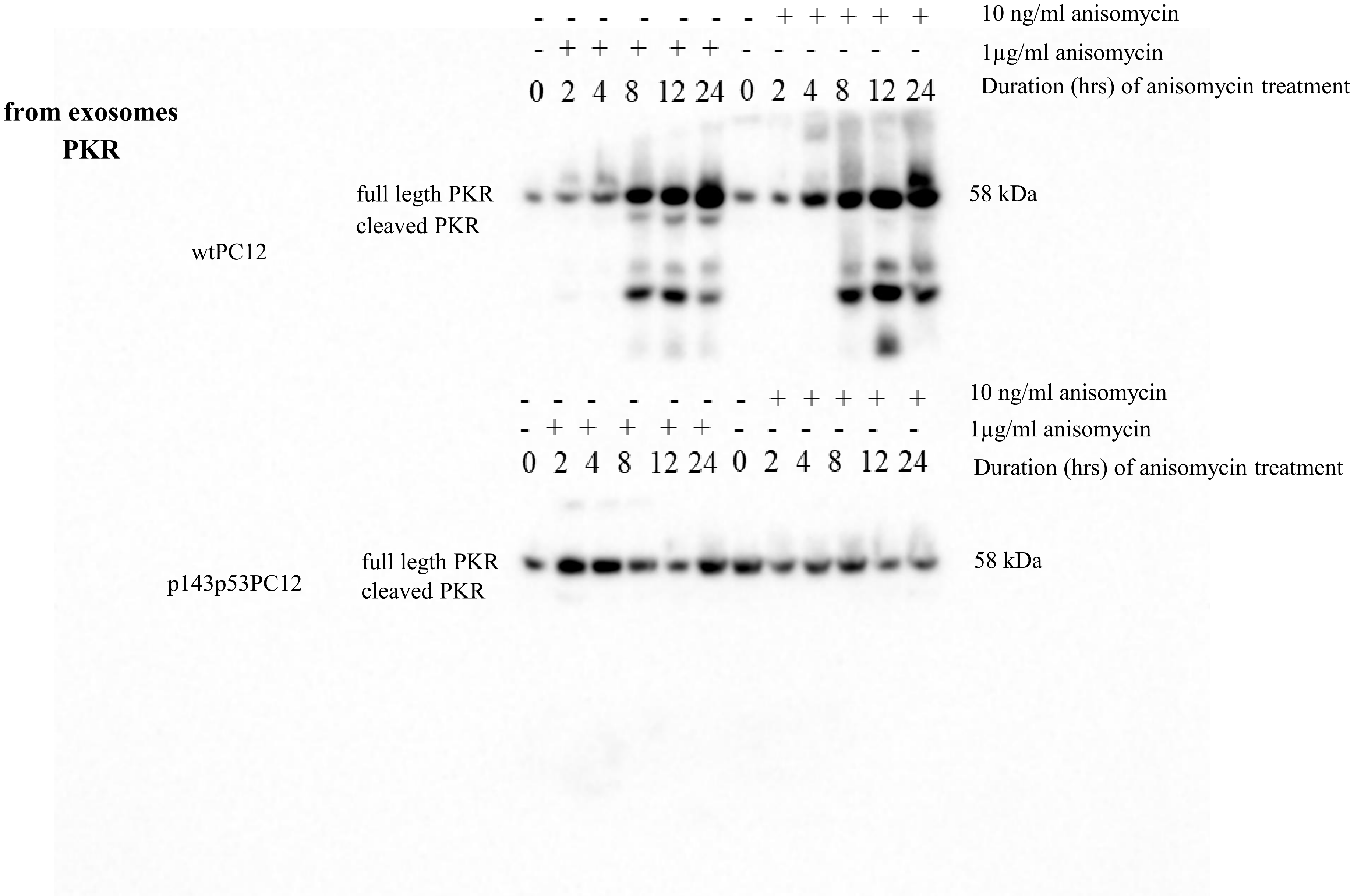

Supplementary Figure 3. Original blots of Figure 3.

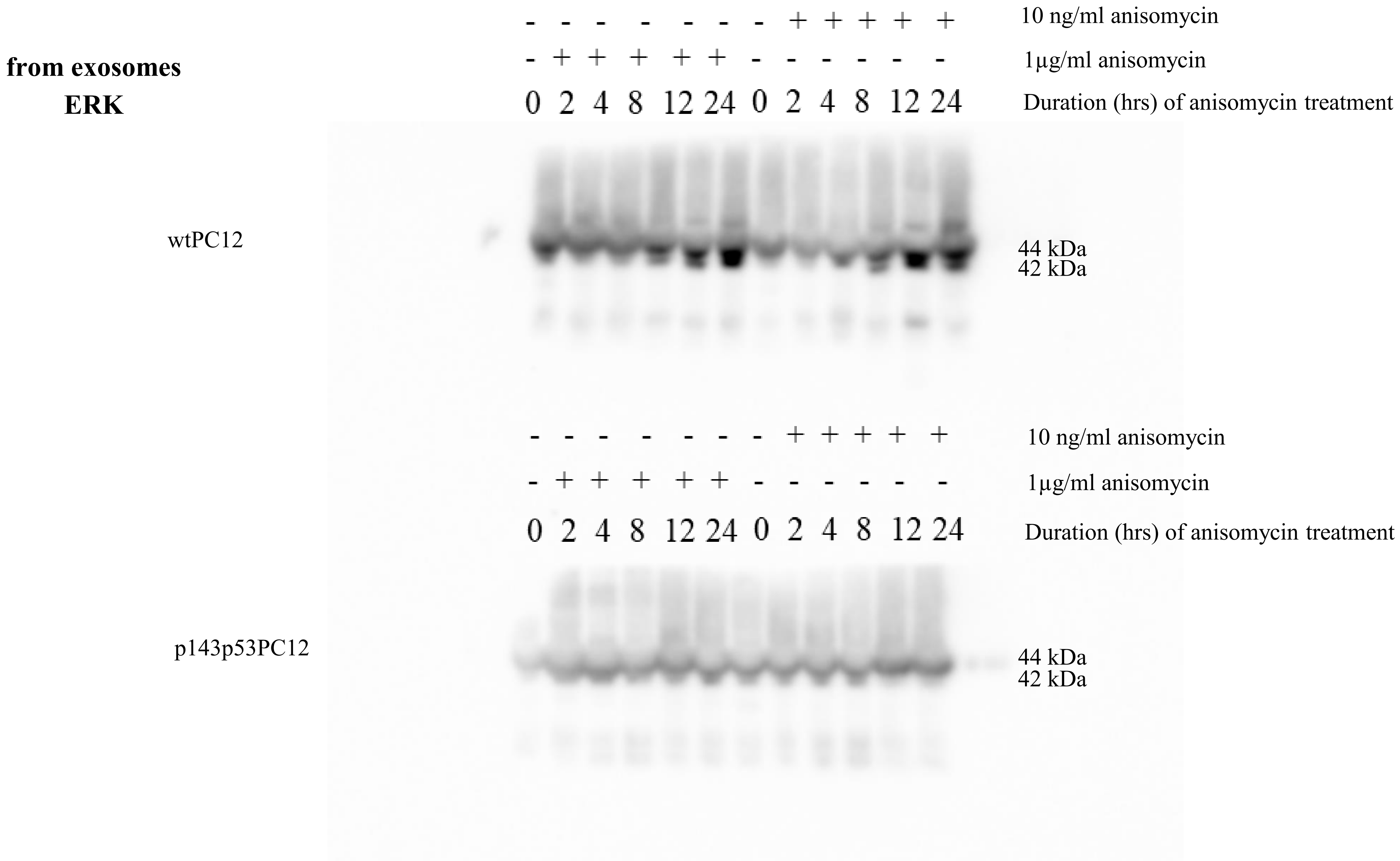

Supplement: Supplementary file 1 [file biology-14-01634-s001.zip › biology-3945588-supplementary/File S1.pdf]
