# Supplementary material for: The Effect of a Dominant Inhibitory p53 Protein on Stress Responses Induced by Toxic and Non-Toxic Concentrations of Anisomycin in PC12 Cells
Source: Biology (Basel). 2025 Nov 21;14(12):1634. doi: 10.3390/biology14121634 (PMC12729296; doi:10.3390/biology14121634)
Supplement: Supplementary file 1 [file biology-14-01634-s001.zip › biology-3945588-supplementary/File S2 Densitometric analysis.pdf]

Figure 1a.

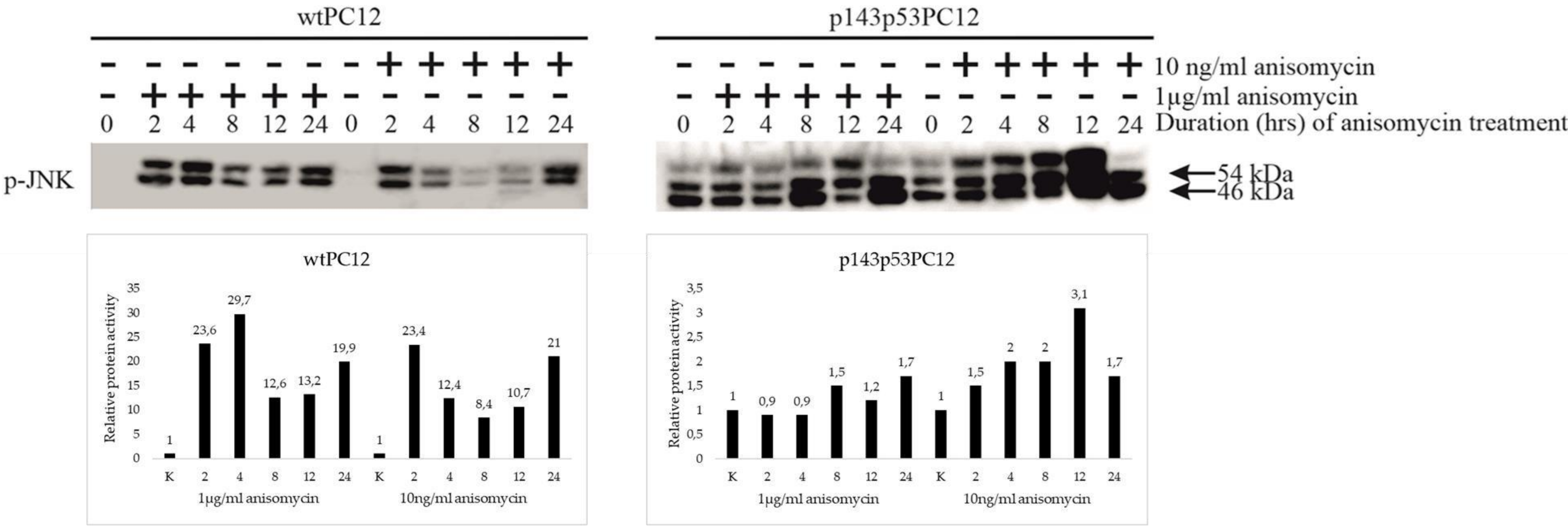
wtPC12
p143p53PC12
10 ng/ml anisomycin
1µg/ml anisomycin
Duration (hrs) of anisomycin treatment
0 2 4 8 12 24 0 2 4 8 12 24
p-JNK
54 kDa
46 kDa
Relative protein activity
1 23,6 29,7 12,6 13,2 19,9 1 23,4 12,4 8,4 10,7 21
1 0,9 0,9 1,5 1,2 1,7 1 1,5 2 2 3,1 1,7
K 2 4 8 12 24 K 2 4 8 12 24
1µg/ml anisomycin
10ng/ml anisomycin

Figure 1a.

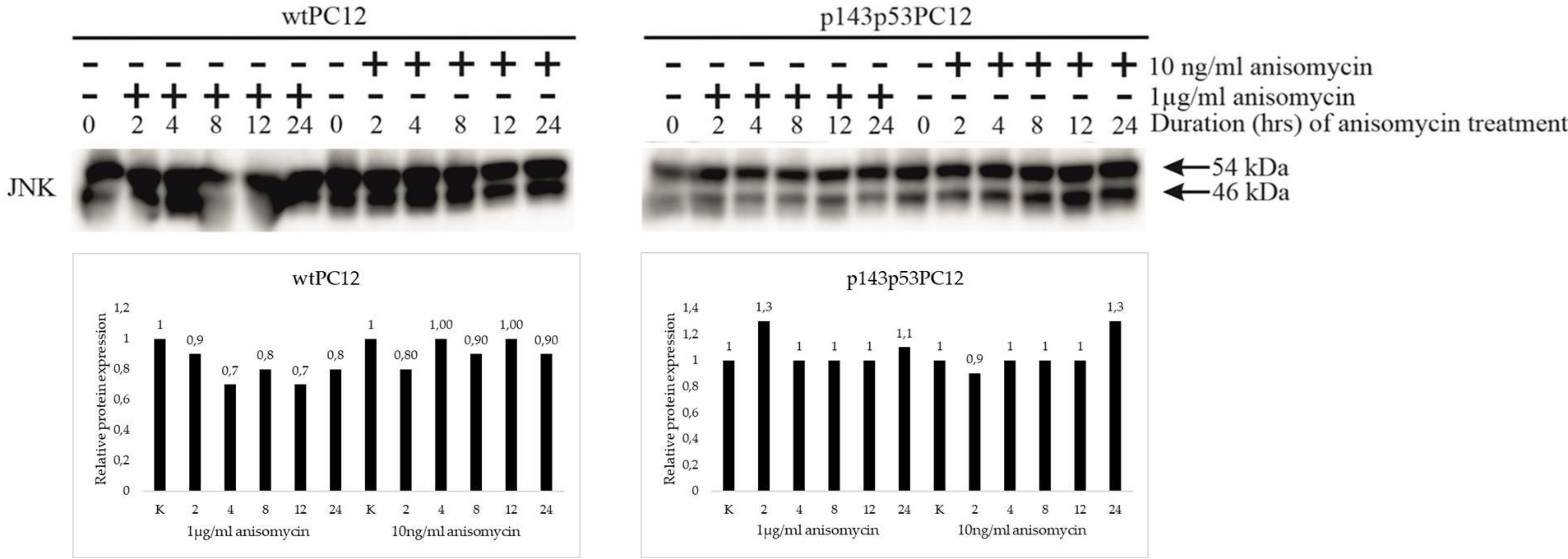
wtPC12
p143p53PC12
10 ng/ml anisomycin
1μg/ml anisomycin
Duration (hrs) of anisomycin treatment
0 2 4 8 12 24 0 2 4 8 12 24
JNK
54 kDa
46 kDa
wtPC12
Relative protein expression
1 0,9 0,7 0,8 0,7 0,8 1 0,80 1,00 0,90 1,00 0,90
K 2 4 8 12 24 K 2 4 8 12 24
1μg/ml anisomycin
10ng/ml anisomycin
p143p53PC12
Relative protein expression
1 1,3 1 1 1 1,1 1 0,9 1 1 1 1,3
K 2 4 8 12 24 K 2 4 8 12 24
1μg/ml anisomycin
10ng/ml anisomycin

Figure 1a.

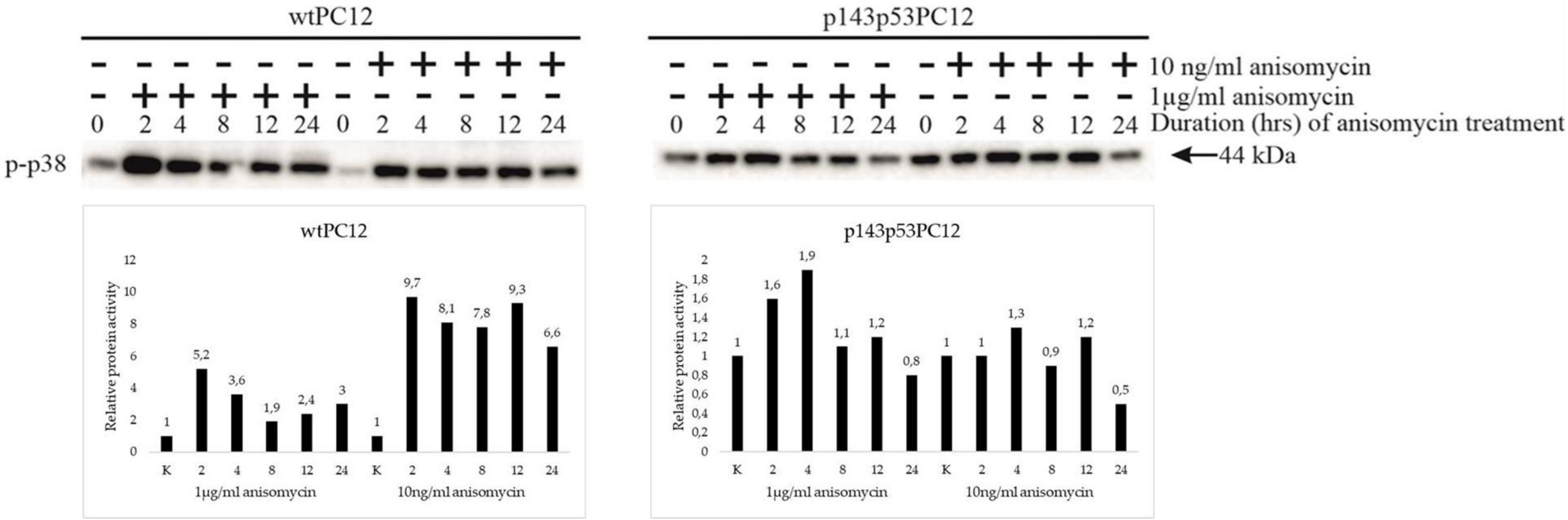
wtPC12
p143p53PC12
10 ng/ml anisomycin
1μg/ml anisomycin
Duration (hrs) of anisomycin treatment
0 2 4 8 12 24 0 2 4 8 12 24
p-p38
44 kDa
wtPC12
Relative protein activity
1 5,2 3,6 1,9 2,4 3 1 9,7 8,1 7,8 9,3 6,6
K 2 4 8 12 24 K 2 4 8 12 24
1μg/ml anisomycin
10ng/ml anisomycin
p143p53PC12
Relative protein activity
1 1,6 1,9 1,1 1,2 0,8 1 1 1,3 0,9 1,2 0,5
K 2 4 8 12 24 K 2 4 8 12 24
1μg/ml anisomycin
10ng/ml anisomycin

Figure 1a.

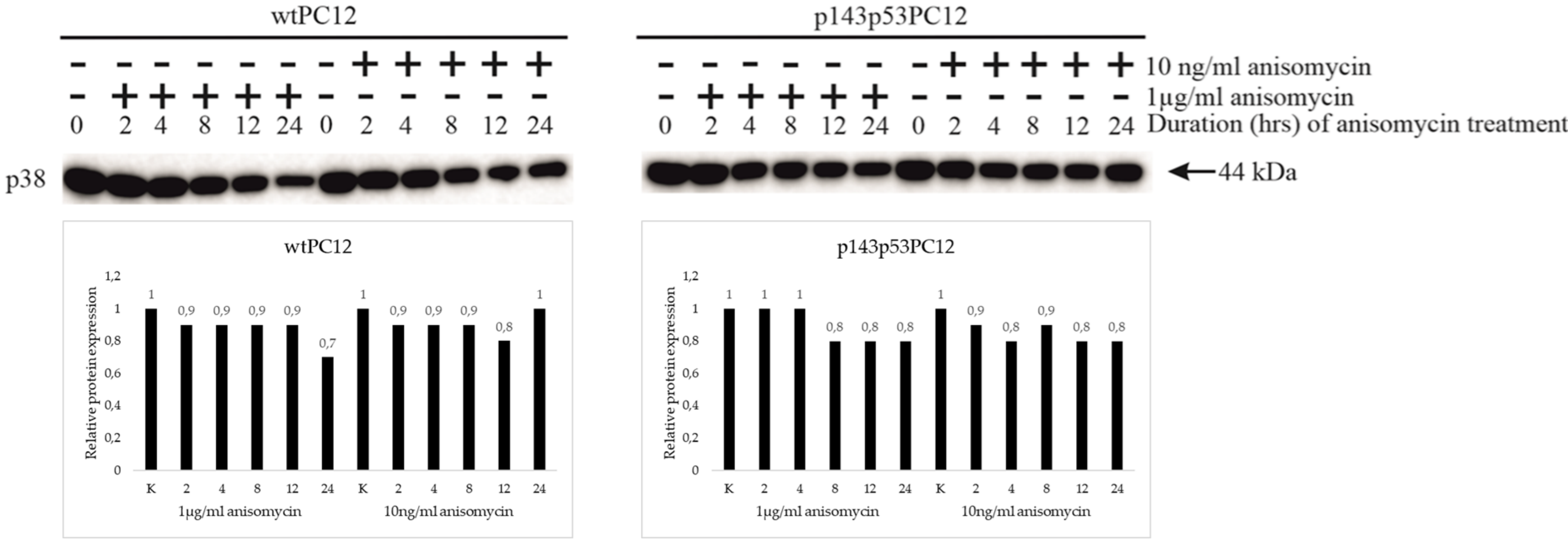
wtPC12
p143p53PC12
10 ng/ml anisomycin
1µg/ml anisomycin
Duration (hrs) of anisomycin treatment
0 2 4 8 12 24 0 2 4 8 12 24
p38
44 kDa
wtPC12
p143p53PC12
Relative protein expression
1,2
1
0,8
0,6
0,4
0,2
0
K 2 4 8 12 24 K 2 4 8 12 24
1µg/ml anisomycin
10ng/ml anisomycin
1 0,9 0,9 0,9 0,9 0,7 1 0,9 0,9 0,9 0,8 1
1 1 1 0,8 0,8 0,8 1 0,9 0,8 0,9 0,8 0,8

Figure 1a.

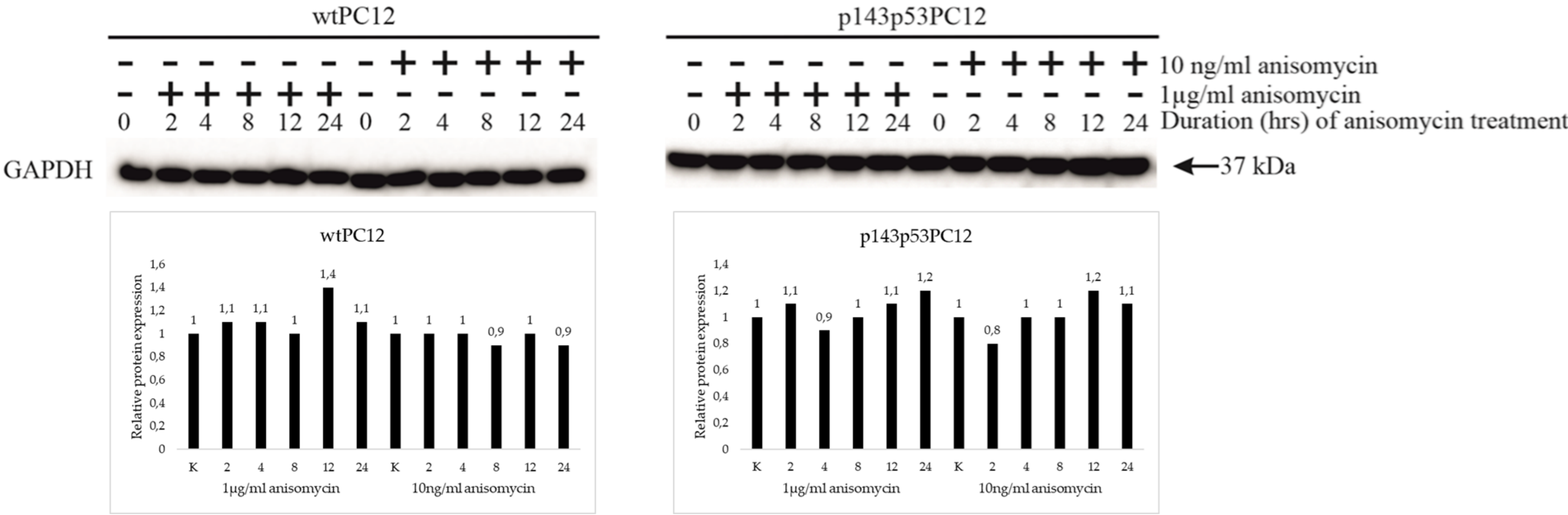

wtPC12
p143p53PC12
10 ng/ml anisomycin
1μg/ml anisomycin
Duration (hrs) of anisomycin treatment
0 2 4 8 12 24 0 2 4 8 12 24
GAPDH
37 kDa
Relative protein expression
1μg/ml anisomycin
10ng/ml anisomycin
K 2 4 8 12 24 K 2 4 8 12 24

Figure 1a.

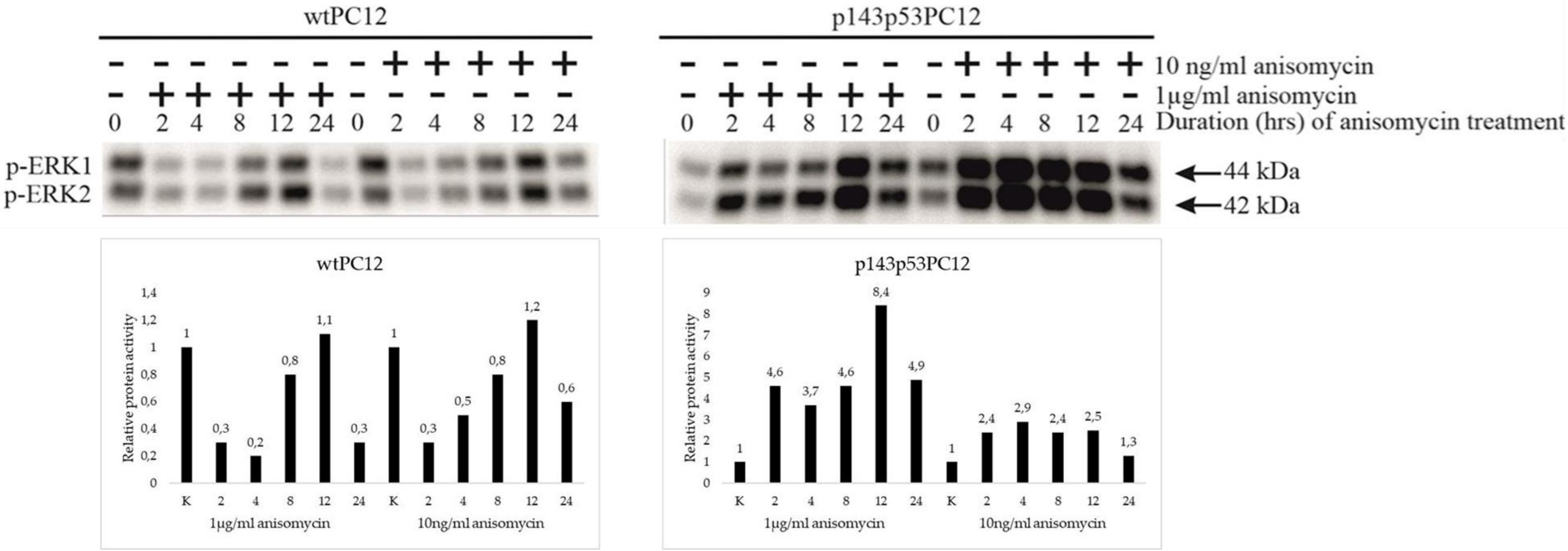
wtPC12
p143p53PC12
10 ng/ml anisomycin
1μg/ml anisomycin
Duration (hrs) of anisomycin treatment
0 2 4 8 12 24 0 2 4 8 12 24
p-ERK1
p-ERK2
44 kDa
42 kDa
wtPC12
Relative protein activity
1,4
1,2
1
0,8
0,6
0,4
0,2
0
1 0,3 0,2 0,8 1,1 0,3 1 0,3 0,5 0,8 1,2 0,6
K 2 4 8 12 24 K 2 4 8 12 24
1μg/ml anisomycin
10ng/ml anisomycin
p143p53PC12
Relative protein activity
9 8 7 6 5 4 3 2 1 0
1 4,6 3,7 4,6 8,4 4,9 1 2,4 2,9 2,4 2,5 1,3
K 2 4 8 12 24 K 2 4 8 12 24
1μg/ml anisomycin
10ng/ml anisomycin

Figure 1a.

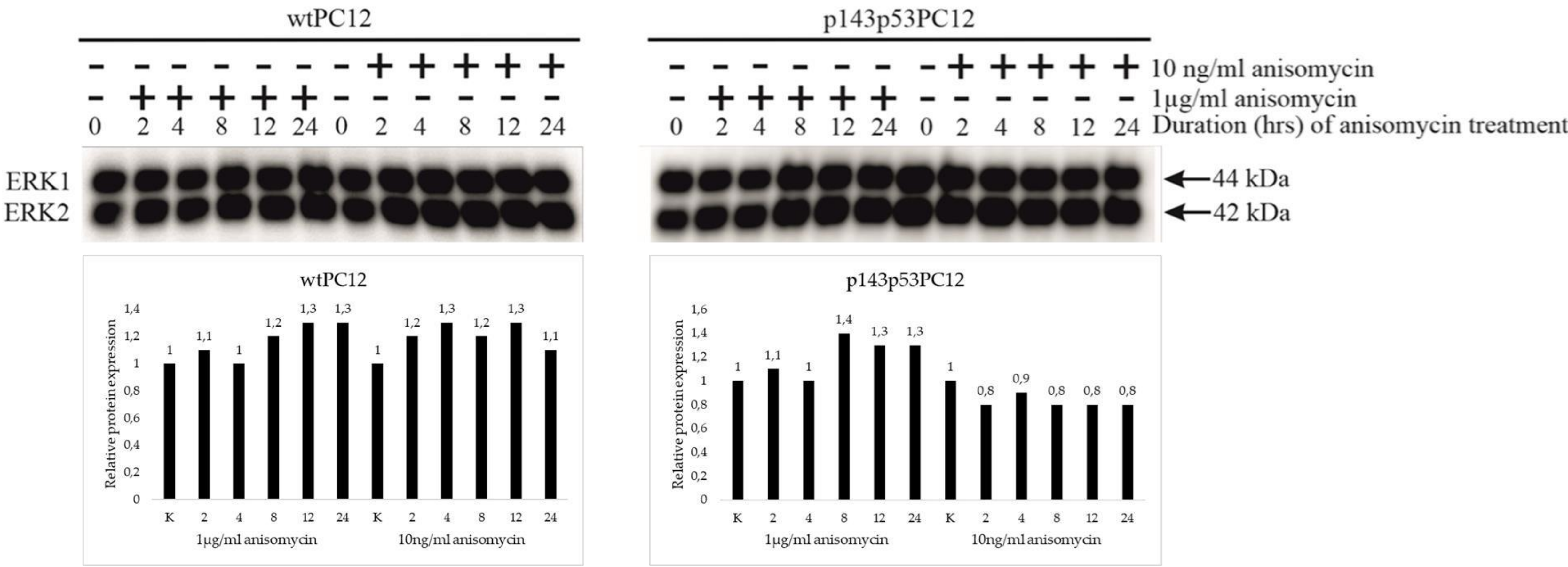
wtPC12
p143p53PC12
10 ng/ml anisomycin
1µg/ml anisomycin
Duration (hrs) of anisomycin treatment
0 2 4 8 12 24 0 2 4 8 12 24
ERK1
ERK2
44 kDa
42 kDa
wtPC12
Relative protein expression
1 1,1 1 1,2 1,3 1,3 1 1,2 1,3 1,2 1,3 1,1
K 2 4 8 12 24 K 2 4 8 12 24
1µg/ml anisomycin
10ng/ml anisomycin
p143p53PC12
Relative protein expression
1 1,1 1 1,4 1,3 1,3 1 0,8 0,9 0,8 0,8 0,8
K 2 4 8 12 24 K 2 4 8 12 24
1µg/ml anisomycin
10ng/ml anisomycin

Figure 1b.

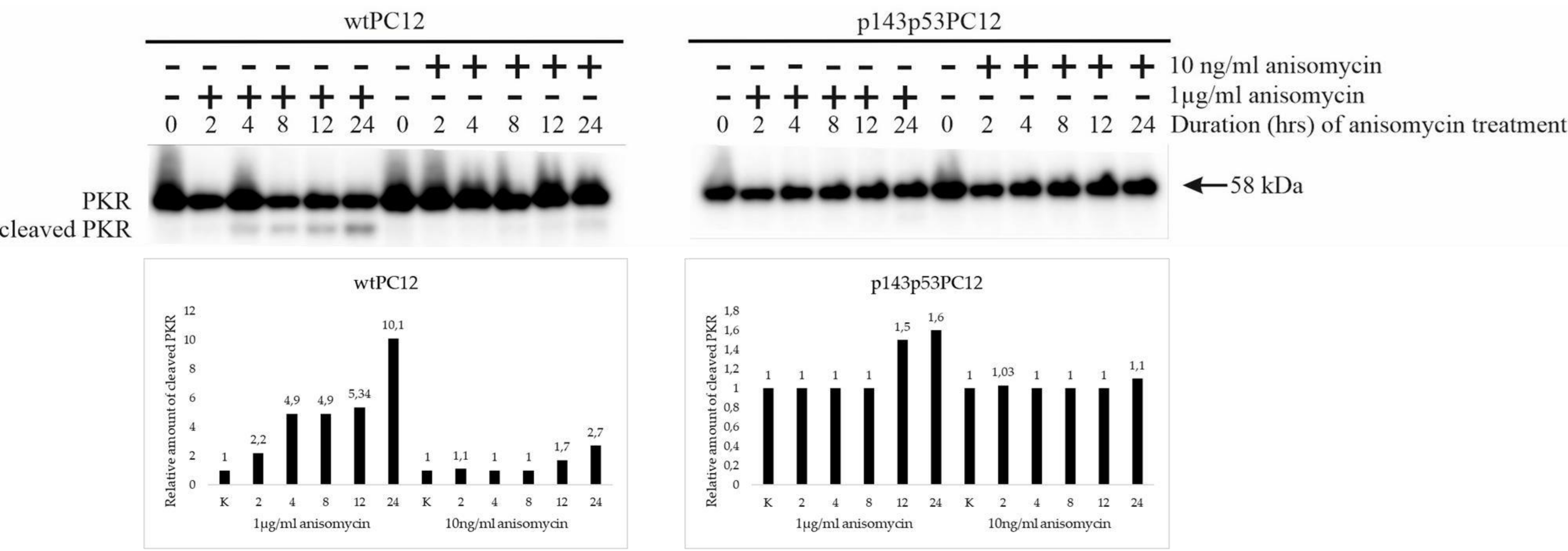
wtPC12
p143p53PC12
10 ng/ml anisomycin
1µg/ml anisomycin
Duration (hrs) of anisomycin treatment
0 2 4 8 12 24 0 2 4 8 12 24
PKR
cleaved PKR
58 kDa
wtPC12
Relative amount of cleaved PKR
1 2,2 4,9 4,9 5,34 10,1 1 1,1 1 1 1,7 2,7
K 2 4 8 12 24 K 2 4 8 12 24
1µg/ml anisomycin
10ng/ml anisomycin
p143p53PC12
Relative amount of cleaved PKR
1 1 1 1 1,5 1,6 1 1,03 1 1 1 1,1
K 2 4 8 12 24 K 2 4 8 12 24
1µg/ml anisomycin
10ng/ml anisomycin

Figure 1b.

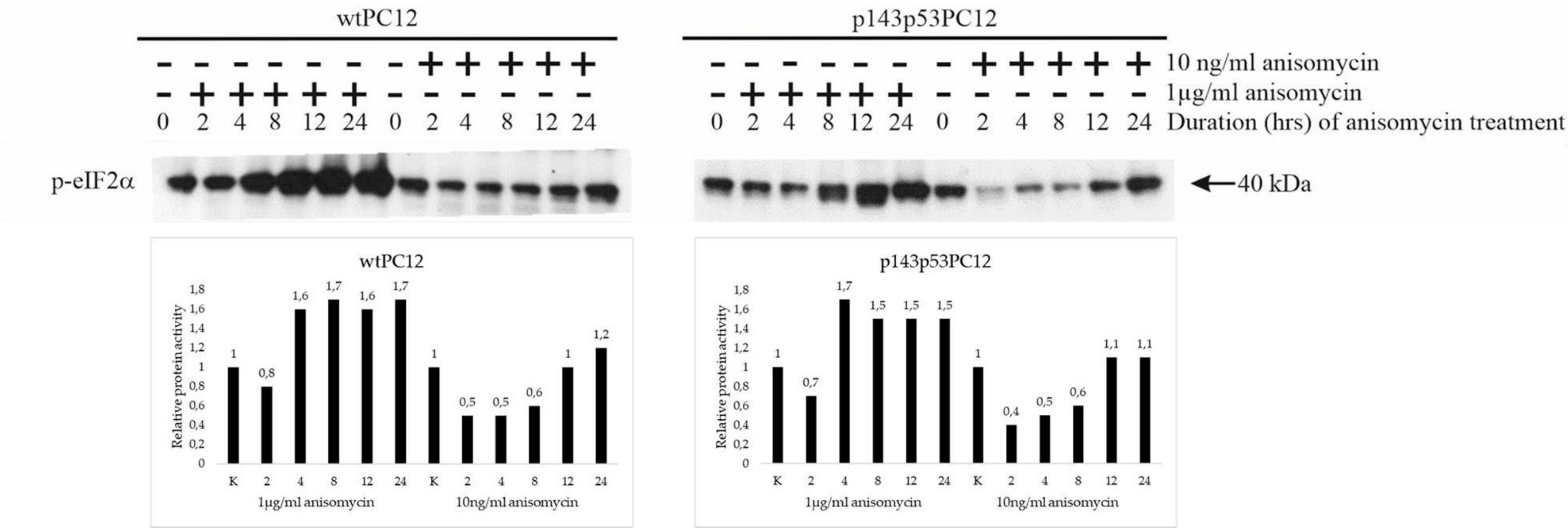
wtPC12
p143p53PC12
10 ng/ml anisomycin
1µg/ml anisomycin
Duration (hrs) of anisomycin treatment
p-eIF2α
40 kDa
Relative protein activity
1µg/ml anisomycin
10ng/ml anisomycin

Figure 1b.

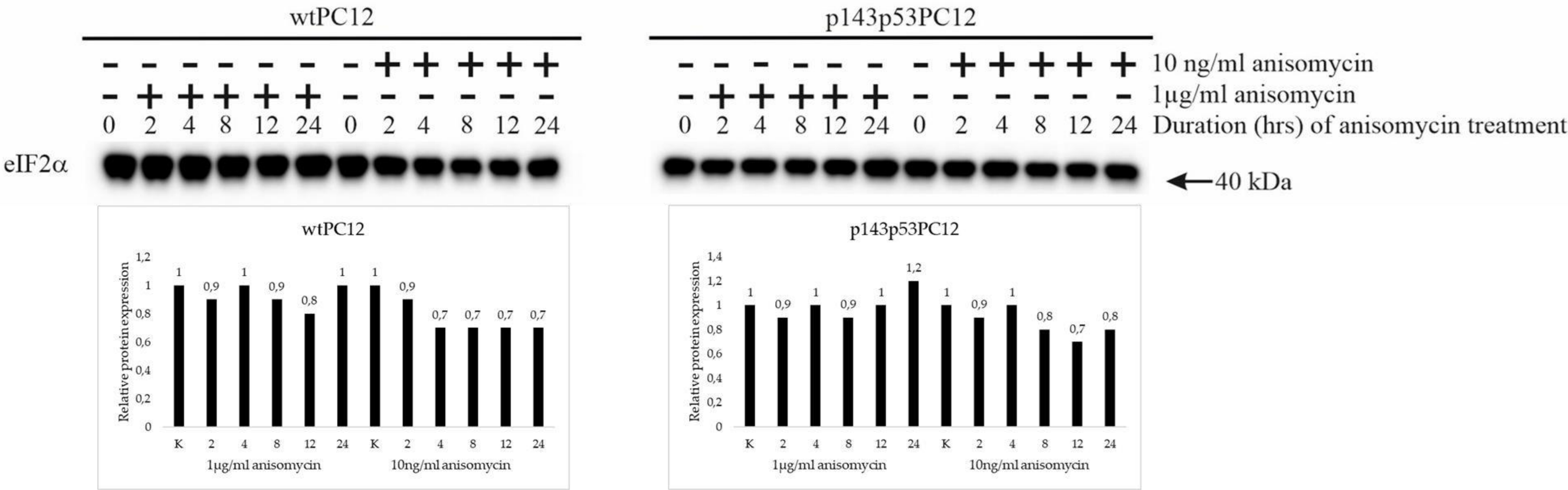
wtPC12
p143p53PC12
10 ng/ml anisomycin
1μg/ml anisomycin
Duration (hrs) of anisomycin treatment
eIF2α
40 kDa
Relative protein expression
1μg/ml anisomycin
10ng/ml anisomycin

Figure 2.

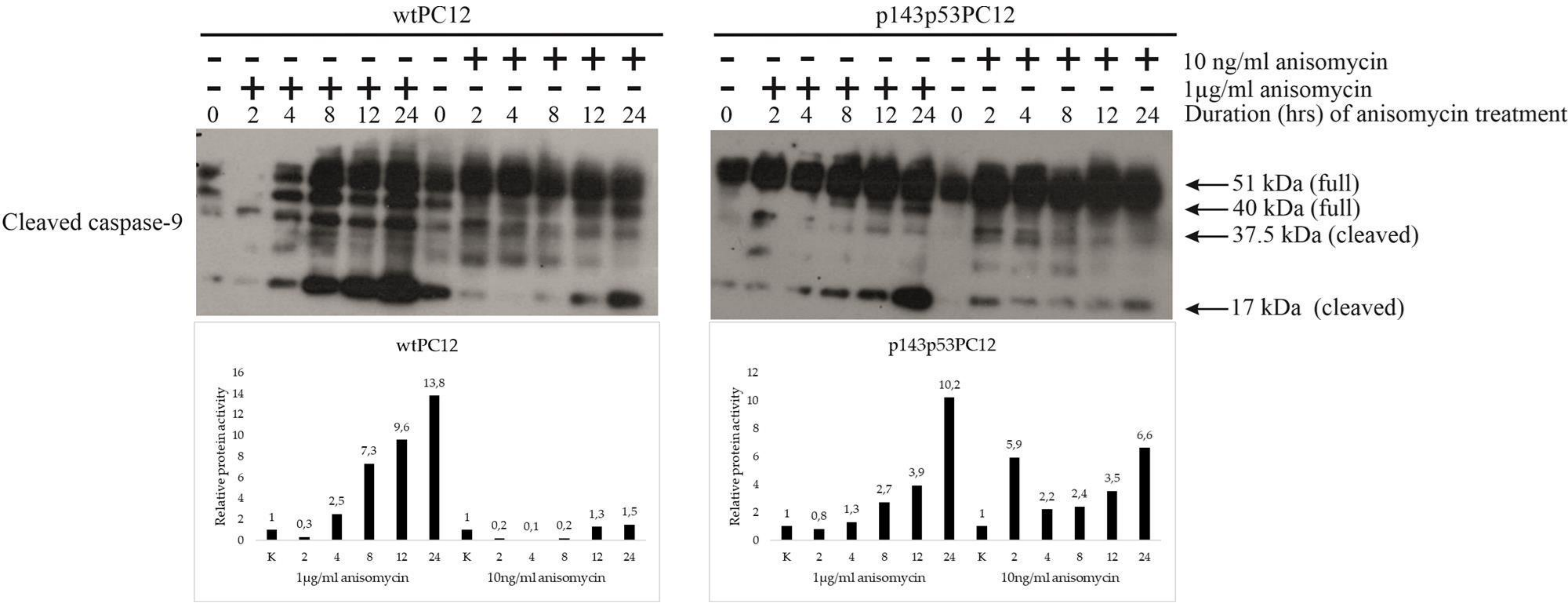
wtPC12
p143p53PC12
10 ng/ml anisomycin
1µg/ml anisomycin
Duration (hrs) of anisomycin treatment
0 2 4 8 12 24 0 2 4 8 12 24
Cleaved caspase-9
51 kDa (full)
40 kDa (full)
37.5 kDa (cleaved)
17 kDa (cleaved)
Relative protein activity
1 0,3 2,5 7,3 9,6 13,8 1 0,2 0,1 0,2 1,3 1,5
1 0,8 1,3 2,7 3,9 10,2 1 5,9 2,2 2,4 3,5 6,6
K 2 4 8 12 24 K 2 4 8 12 24
1µg/ml anisomycin
10ng/ml anisomycin

Figure 2.

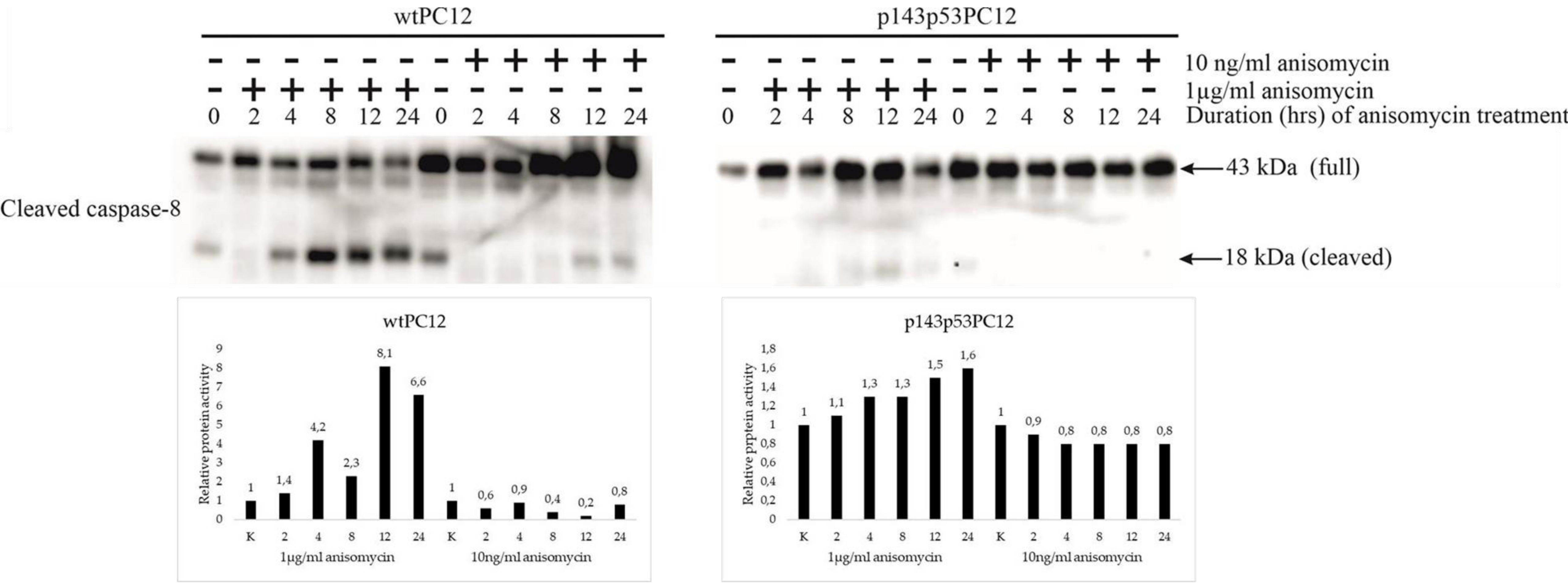
wtPC12
p143p53PC12
10 ng/ml anisomycin
1μg/ml anisomycin
Duration (hrs) of anisomycin treatment
0 2 4 8 12 24 0 2 4 8 12 24
Cleaved caspase-8
43 kDa (full)
18 kDa (cleaved)
wtPC12
Relative protein activity
1 1,4 4,2 2,3 8,1 6,6 1 0,6 0,9 0,4 0,2 0,8
K 2 4 8 12 24 K 2 4 8 12 24
1μg/ml anisomycin
10ng/ml anisomycin
p143p53PC12
Relative prptein activity
1 1,1 1,3 1,3 1,5 1,6 1 0,9 0,8 0,8 0,8 0,8
K 2 4 8 12 24 K 2 4 8 12 24
1μg/ml anisomycin
10ng/ml anisomycin

Figure 2.

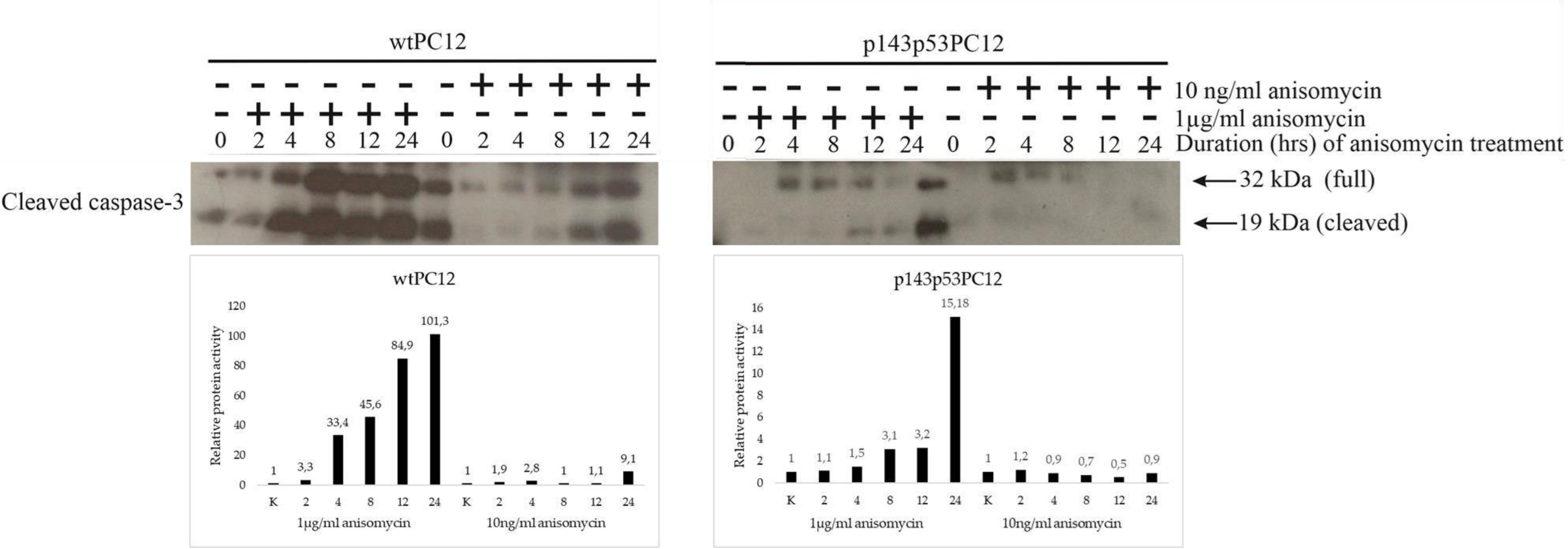
wtPC12
p143p53PC12
10 ng/ml anisomycin
1µg/ml anisomycin
Duration (hrs) of anisomycin treatment
0 2 4 8 12 24 0 2 4 8 12 24
Cleaved caspase-3
32 kDa (full)
19 kDa (cleaved)
Relative protein activity
1 3,3 33,4 45,6 84,9 101,3 1 1,9 2,8 1 1,1 9,1
1 1,1 1,5 3,1 3,2 15,18 1 1,2 0,9 0,7 0,5 0,9
K 2 4 8 12 24 K 2 4 8 12 24
1µg/ml anisomycin
10ng/ml anisomycin

Figure 2.

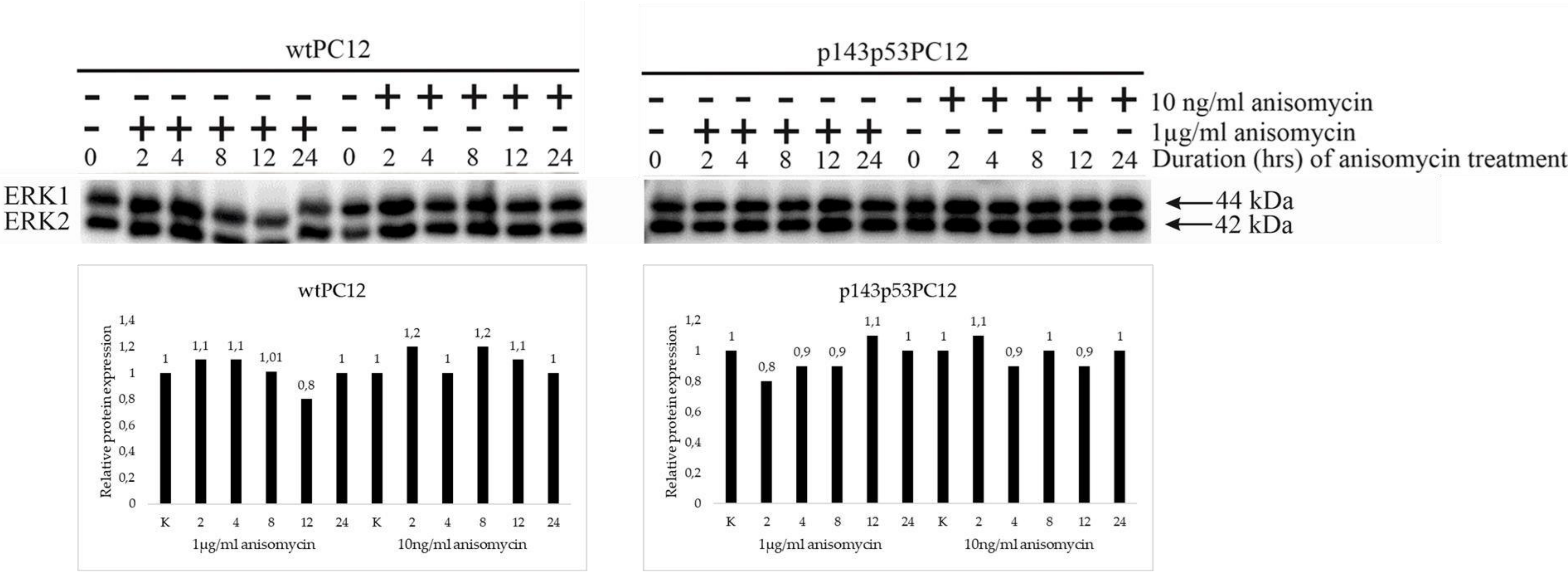

wtPC12
p143p53PC12
10 ng/ml anisomycin
1µg/ml anisomycin
Duration (hrs) of anisomycin treatment
0 2 4 8 12 24 0 2 4 8 12 24
ERK1
ERK2
44 kDa
42 kDa
Relative protein expression
K 2 4 8 12 24 K 2 4 8 12 24
1µg/ml anisomycin
10ng/ml anisomycin
1 1,1 1,1 1,01 0,8 1 1 1,2 1 1,2 1,1 1
1 0,8 0,9 0,9 1,1 1 1 1,1 0,9 1 0,9 1

Figure 3a.

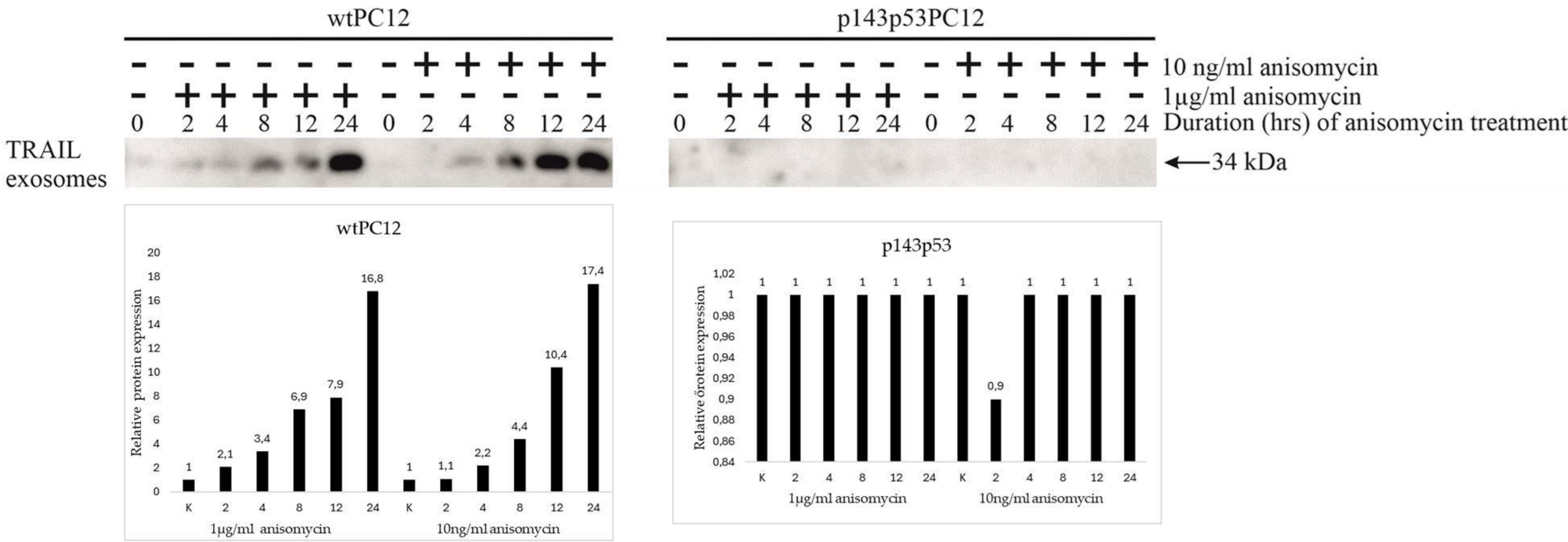

wtPC12
p143p53PC12
10 ng/ml anisomycin
1µg/ml anisomycin
Duration (hrs) of anisomycin treatment
0 2 4 8 12 24 0 2 4 8 12 24
TRAIL exosomes
34 kDa
wtPC12
Relative protein expression
1 2,1 3,4 6,9 7,9 16,8 1 1,1 2,2 4,4 10,4 17,4
K 2 4 8 12 24 K 2 4 8 12 24
1µg/ml anisomycin
10ng/ml anisomycin
p143p53
Relative ŕrotein expression
1 1 1 1 1 1 1 0,9 1 1 1 1

Figure 3a.

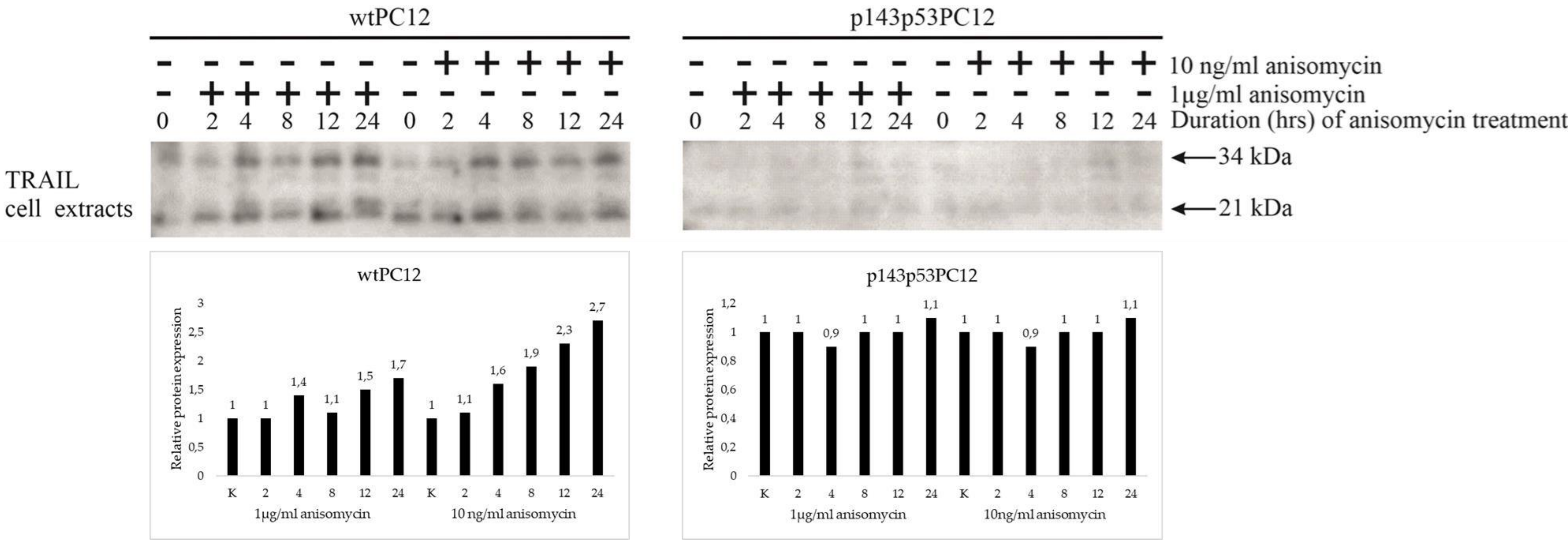
wtPC12
p143p53PC12
10 ng/ml anisomycin
1µg/ml anisomycin
Duration (hrs) of anisomycin treatment
0 2 4 8 12 24 0 2 4 8 12 24
TRAIL
cell extracts
34 kDa
21 kDa
wtPC12
Relative protein expression
1 1 1,4 1,1 1,5 1,7 1 1,1 1,6 1,9 2,3 2,7
K 2 4 8 12 24 K 2 4 8 12 24
1µg/ml anisomycin
10 ng/ml anisomycin
p143p53PC12
Relative protein expression
1 1 0,9 1 1 1,1 1 1 0,9 1 1 1,1
K 2 4 8 12 24 K 2 4 8 12 24
1µg/ml anisomycin
10ng/ml anisomycin

Figure 3b.

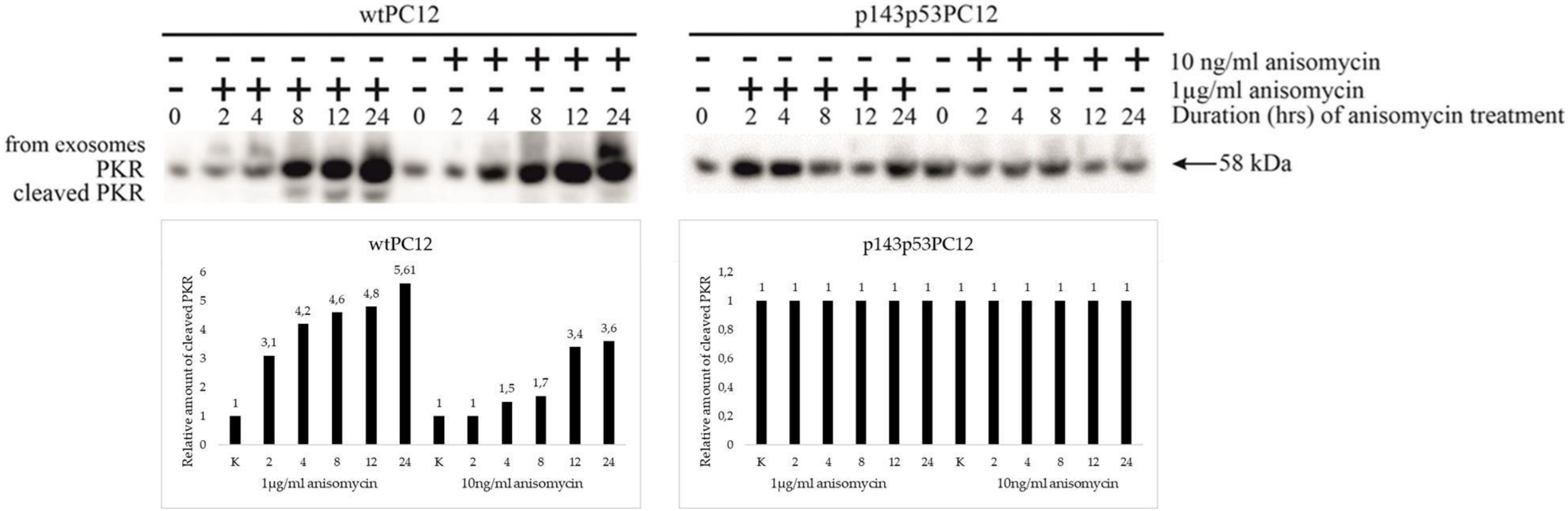
wtPC12
p143p53PC12
10 ng/ml anisomycin
1μg/ml anisomycin
Duration (hrs) of anisomycin treatment
from exosomes
PKR
cleaved PKR
58 kDa
Relative amount of cleaved PKR
1μg/ml anisomycin
10ng/ml anisomycin
K
1
3,1
4,2
4,6
4,8
5,61
1,5
1,7
3,4
3,6

Figure 3b.

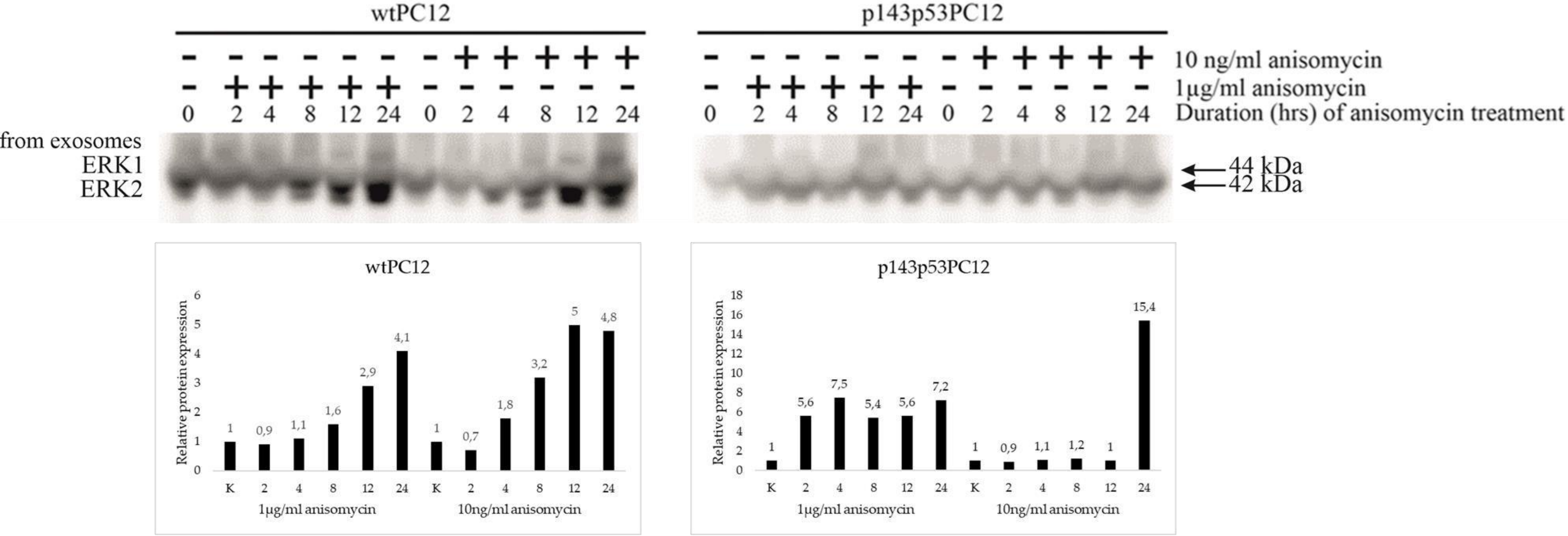
wtPC12
p143p53PC12
10 ng/ml anisomycin
1µg/ml anisomycin
Duration (hrs) of anisomycin treatment
0 2 4 8 12 24 0 2 4 8 12 24
from exosomes
ERK1
ERK2
44 kDa
42 kDa
wtPC12
Relative protein expression
1 0,9 1,1 1,6 2,9 4,1 1 0,7 1,8 3,2 5 4,8
K 2 4 8 12 24 K 2 4 8 12 24
1µg/ml anisomycin
10ng/ml anisomycin
p143p53PC12
Relative protein expression
1 5,6 7,5 5,4 5,6 7,2 1 0,9 1,1 1,2 1 15,4
K 2 4 8 12 24 K 2 4 8 12 24
1µg/ml anisomycin
10ng/ml anisomycin
